# Intracellular XBP1-IL-24 axis dismantles cytotoxic unfolded protein response in the liver

**DOI:** 10.1038/s41419-019-2209-6

**Published:** 2020-01-06

**Authors:** Jianye Wang, Bian Hu, Zhicong Zhao, Haiyan Zhang, He Zhang, Zhenjun Zhao, Xiong Ma, Bin Shen, Beicheng Sun, Xingxu Huang, Jiajie Hou, Qiang Xia

**Affiliations:** 1 Department of Liver Surgery, Renji Hospital, School of Medicine, Shanghai Jiaotong University, Shanghai, China; 2 School of Life Science and Technology, ShanghaiTech University, Shanghai, China; 3 State Key Laboratory of Oncology in South China, Collaborative Innovation Center for Cancer Medicine, Sun Yat-sen University Cancer Center, Guangzhou, China; 4 Department of Surgery, The University of Hong Kong-Shenzhen Hospital, Shenzhen, China; 5 Division of Gastroenterology and Hepatology, Key Laboratory of Gastroenterology and Hepatology, Ministry of Health, State Key Laboratory for Oncogenes and Related Genes, Renji Hospital, School of Medicine, Shanghai Jiao Tong University, and Shanghai Institute of Digestive Disease, Shanghai, China; 6 State Key Laboratory of Reproductive Medicine, Department of Histology and Embryology, Nanjing Medical University, Nanjing, China; 7 Department of Hepatobiliary Surgery, The Affiliated Drum Tower Hospital of Nanjing University Medical School, Nanjing, China; 8 Department of Liver Surgery, Sun Yat-sen University Cancer Center, Guangzhou, China

**Keywords:** Cell biology, Liver diseases

## Abstract

Endoplasmic reticulum (ER) stress-associated cell death is prevalent in various liver diseases. However, the determinant mechanism how hepatocytes survive unresolved stress was still unclear. Interleukin-24 (IL-24) was previously found to promote ER stress-mediated cell death, and yet its expression and function in the liver remained elusive. Here we identified an antiapoptotic role of IL-24, which transiently accumulated within ER-stressed hepatocytes in a X-box binding protein 1 (XBP1)-dependent manner. Disruption of IL-24 increased cell death in the CCL_4_- or APAP-challenged mouse liver or Tm-treated hepatocytes. In contrast, pharmaceutical blockade of eukaryotic initiation factor 2α (eIF2α) or genetical ablation of C/EBP homologous protein (CHOP) restored hepatocyte function in the absence of IL-24. In a clinical setting, patients with acute liver failure manifested a profound decrease of hepatic IL-24 expression, which was associated with disease progression. In conclusion, intrinsic hepatocyte IL-24 maintains ER homeostasis by restricting the eIF2α-CHOP pathway-mediated stress signal, which might be exploited as a bio-index for prognosis or therapeutic intervention in patients with liver injury.

## Introduction

The liver, one of the most vital organs in metabolic homeostasis, has a unique potential to fully recover from acute liver injury. Despite recent studies elucidating various molecular pathways involved in liver damage^1^, a further understanding of the pivotal life-and-death decision mechanism is needed to improve current therapeutics. Endoplasmic reticulum (ER) content is rich in hepatocytes and participates in the processes of synthesizing, folding and trafficking of proteins^2,3^. Environmental stimuli or nutrient fluctuations disrupt the ER protein-folding procedure, referred to as ER stress^4^. With an accumulation of misfolded proteins in ER lumen, the unfolded protein response (UPR), a collection of intracellular signal pathways, is activated to increase protein-folding capacity and reduce global protein synthesis. Once the molecular adaption fails in resolving the protein-folding defect, hepatocytes enter persistent ER stress, which results in apoptosis^5^. ER stress-related apoptosis has been found in fatty liver disease, viral hepatitis, and alcohol or drug-induced liver injury^3,6,7^. The transcription factor C/EBP homologous protein (CHOP) mediates the most well-characterized proapoptotic pathway resulted from unresolved ER stress. CHOP induces the expression of proapoptotic BH3-only protein Bim, the cell surface death receptor TRAIL receptor 2, and inhibits Bcl2 transcription^8–11^. As previously reported, CHOP-deficient mice were protected from acetaminophen (APAP)-induced liver damage and conferred a survival advantage^12^.

Interleukin-24 (IL-24) was first identified as a negative regulator in human melanoma^13,14^. As an IL-10 superfamily member, IL-24 has been reported to exert a bystander anti-cancer function, but has no deleterious effect toward noncancerous cell^13,15–17^. Like other secretory proteins, IL-24 precursor, which is 206 amino acids in length, translocates to the ER lumen before it proceeds to the secretory pathway. Independently of its cognate receptors, adenovirus-mediated IL-24 overexpression in melanoma cells led to induction of apoptosis by interaction with glucose-regulated protein 78 (GRP78) and upregulation of GADD family genes, including CHOP^18,19^. Nonetheless, little is known about IL-24 expression and its correlation with ER stress in noncancerous cells. Interestingly, IL-24 production was elevated in diabetic pancreatic islets, where it induced beta cell ER stress and impaired glucose tolerance^20^. But it remains unclear whether IL-24 adapts ER homeostasis in epithelial cells.

Given the abundant IL-24 expression in the normal mouse or human liver detected in our preliminary experiments, the role of hepatocyte IL-24 in liver diseases has yet to be deciphered. To search for a possible link between IL-24 and ER stress within hepatocytes, we employed two mouse models characterizing IL-24 in the duration of acute liver injury. Unexpectedly, IL-24 deficiency did not alleviate liver damage but sensitized ER stressed hepatocytes to death. By monitoring tunicamycin (Tm)-stimulated mouse hepatocytes in vitro or manipulating the IL-24 level or UPR pathway in vivo, we further confirmed antiapoptotic function of intracellular IL-24. Indeed, we revealed that hepatocyte IL-24 governs the intrinsic adaption to ER stress by control of PERK-eIF2α-CHOP pathway. Collectively, these results highlight profound implications for understanding hepatocyte ER homeostasis and identify IL-24 as a critical anti-stress factor in the liver.

## Results

### Hepatocyte IL-24 transiently increases during ER stress-related acute liver injury

**Fig. 1:**
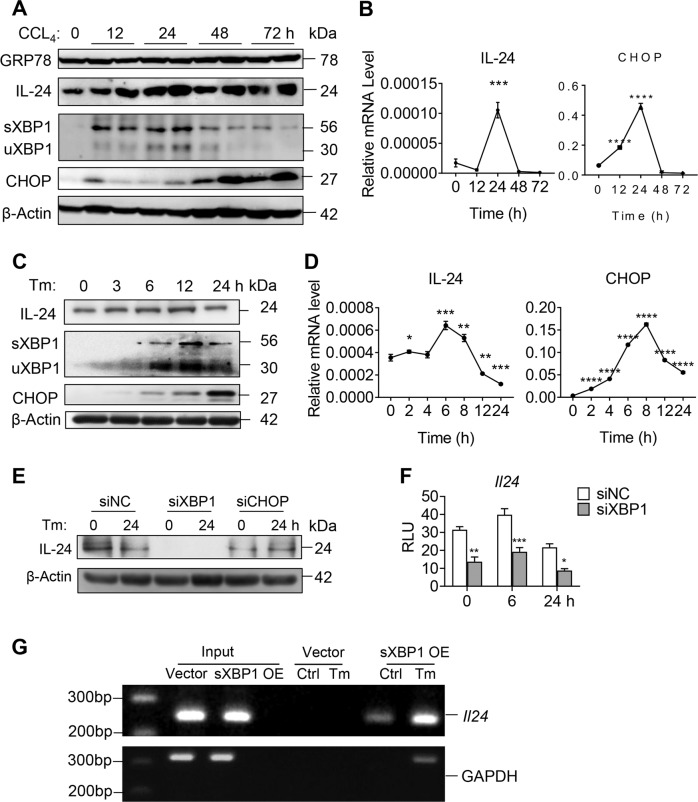
IL-24 expression in the ER stressed-mouse liver and hepatocytes. **a**, **b** WT mice were intraperitoneally injected with a single dose of CCL_4_ (2 ml/kg). **a** GRP78, IL-24 and CHOP protein levels in the liver tissues were evaluated representatively by western blot at indicated time points. Results are normalized to β-actin. *n* *=* 3 independent experiments. **b** IL-24 and CHOP mRNA levels in the liver tissues were assessed by qRT-PCR at indicated time points. Results are normalized to β-actin. *n* *=* 3 biological replicates. AML12 cells were exposed to Tm (5 µg/ml) for the indicated time period. IL-24 and CHOP levels were evaluated by western blot (**c**) and qRT-PCR (**d**) at indicated time points. *n* *=* 2 independent experiments (**c**) or three biological replicates (**d**). **e** AML12 cells were transfected with indicated siRNAs or negative control (NC) 24 h prior to Tm treatment. IL-24 protein levels at indicated time points were assessed by western blot. *n* *=* 3 independent experiments. **f** *Il24* promoter activity in AML12 cells expressing indicated siRNAs, as quantified using luciferase assay. *Renilla* luciferase activity was normalized to firefly activity and presented as relative luciferase activity. *n* = 3 biological replicates. **g** ChIP analysis of the *Il24* promoter in Vector and sXBP1 overexpressing cells (Tm 0 h and 6 h) using flag antibodies. Data are presented as means ± SEM. *P < 0.05, **P < 0.01, ***P < 0.001, ****P *<* 0.0001. *P* values were determined by two-tailed *t* test.

First, we detected the expression of IL-24 among different organs in normal wild type (WT) mice and found it was most highly expressed in the liver (Supplementary Fig. 1A). To explore whether liver IL-24 is linked to ER stress, we treated WT mice with a single dose of CCL_4_ (2 ml/kg) as reported previously^19^. Serum levels of alanine aminotransferase (ALT) and aspartate aminotransferase (AST) were markedly elevated and peaked at 48 h post treatment, then returned to baseline at 72 h time point (Supplementary Fig. 1B). In context, exposure to CCL_4_ unaffected the ER chaperon GRP78 but tremendously evoked CHOP expression in the liver (Fig. 1a, b). Noticeably, IL-24 mRNA level was transiently increased at 24 h, then decreased and reverted to normal level 72 h post CCL_4_ administration. A same trend was observed in IL-24 protein level (Fig. 1a). Regarding the potential inflammatory responses caused by CCL_4_, we also measured the serum IL-24 protein level. Nonetheless, CCL_4_-treated mice exhibited undetectable serum IL-24, which was comparable to that in none-treated WT or IL-24 KO mice (data not shown). Given the fact that hepatocytes take up the majority of hepatic cells, we asked whether the fluctuation of IL-24 expression was occurred in ER stressed hepatocytes. To answer this question, we investigated IL-24 expression in murine hepatocyte cell line AML12 in the presence of an ER stress inducer Tm. Consistent with the in vivo observation, a transient increase of IL-24 level was recapitulated in AML12 upon ER stress, as accompanied by accumulating CHOP expression (Fig. 1c, d). These results suggested a potential role of nonsecreted IL-24 and prompted us to understand how IL-24 was involved in hepatocyte ER stress.

The transcription factors activating transcription factor 4 (ATF4), ATF6, sliced X-box binding protein 1 (sXBP1) and CHOP regulate UPR-related gene expression^5^. Like CHOP, other three molecules were also upregulated in the CCL_4_-exposed mouse liver or ER stressed-AML12 cells, among which sXBP1 was the first to peak (Supplementary Fig. 1C, D). Intriguingly, the murine *Il24* promoter harbors conserved binding motifs for ATF6/XBP1 and CHOP^21,22^(Supplementary Fig. 2A). To explore how IL-24 expression was affected in response to ER stress, we transfected AML12 cells with small interfering RNAs (siRNAs) targeting ATF4, ATF6, XBP1 and CHOP prior to Tm stimulation. IL-24 mRNA levels in these siRNA-expressing cells all decreased as compare to the negative control (NC) (Supplementary Fig. 2B), suggesting a regulatory relation between hepatocyte IL-24 and UPR pathways. Importantly, siXBP1 most significantly inhibited IL-24 mRNA level and blocked its upregulation in response to ER stress. Silencing of XBP1 (but not CHOP or ATF6) repressed IL-24 protein level as well as *Il24 promoter* activity in Tm-stimulated AML12 cells (Fig. 1e, f and Supplementary Fig. 2C). Besides, ChIP assays showed sXBP1 binding to *Il24* promoter in AML12 cells at a basal level, which could be further enhanced by Tm treatment (Fig. 1g), Furthermore, we isolated primary hepatocytes from conditional XBP1 KO (Xbp1^f/w^;Alb^Cre^) mice. XBP1 depletion unaffected cell viability under ER stress, but reduced IL-24 in both mRNA and protein levels (Supplementary Fig. 2D–F).

### Hepatocyte IL-24 deficiency promotes ER stress-related liver injury

**Fig. 2:**
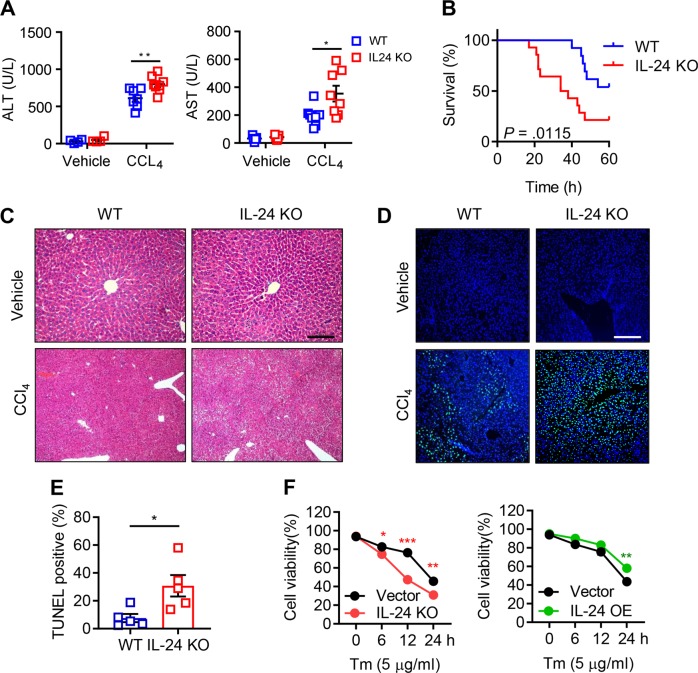
The protective role of hepatocyte IL-24 in ER stress-induced cell death. Sex- and age-matched WT and IL-24-null mice were intraperitoneally injected with vehicle or CCL_4_, randomly. **a** Mouse liver function was assessed by measuring serum ALT (left) and AST (right) levels. *n* *=* 5–8. **b** Mouse survival rate after CCL_4_-treatment was determined via Log-rank (Mantel–Cox) analysis. *n* *=* 11–13. Representatively H&E (**c**) and TUNEL (**d**) staining of the liver tissues from vehicle or CCL_4_-treated mice. *n* *=* 5–8 mice. Scale bar, 100 µm. **e** Hepatocyte apoptosis after CCL_4_-treatment was assessed by counting TUNEL positive cells. *n* *=* 5–8 mice. **f** AML12 cells were transfected with lentiviral vectors expressing IL-24-targeted sgRNA (left, referred to as IL-24 KO) or IL-24 cDNA (right, referred to as IL-24 OE). An empty vector was transfected as a negative control, respectively. Cell viability of indicated AML12 cells after Tm stimulation was assessed by CCK8 assay. *n* = 3 biological replicates. Data are presented as means ± SEM. *P < 0.05, **P < 0.01, ***P < 0.001. *P* values were determined by two-tailed *t* test.

To further dissect the underlying impact of IL-24 fluctuation, IL-24 KO mice, in which 3306 bp of IL-24 allele was depleted, were subjected to CCL_4_-induced liver injury. In comparison to the WT mice, IL-24-null littermates were more susceptible to CCL_4_-induced liver injury, exhibiting a relatively higher ALT and AST level and a lower survival rate (Fig. 2a, b). The exacerbated liver damage in IL-24-null mice was visualized by hematoxylin and eosin (H&E). Meanwhile, a marked increase in the percentage of terminal deoxynucleotidyl transferase dUTP nick end labeling (TUNEL)-positive hepatocyte was observed in IL-24-null mice with respect to WT mice (Fig. 2c, d). Besides, proliferating cell nuclear antigen staining showed an increase of cell proliferation in IL-24 KO mice (Supplementary Fig. 3A), possibly due to compensatory liver regeneration. We then checked the inflammatory status and detected higher IL1A and IL6 and lower TNFA mRNA expression in the IL-24-deficient mouse liver (Supplementary Fig. 3B). Overdose of APAP, an analgesic and antipyretic drug, is the leading cause of drug-induced acute liver injury^23^. Accordingly, we subjected IL-24-null mice to oral administration of APAP, which was evident for inducing ER stress-related liver damage^12^. In context, worse liver function and survival rate and extensive hepatocyte death were manifested in IL-24-deficient group (Supplementary Fig. 3C–E). Given the possibility that the extracellular IL-24 might be implicated in liver injury, we treated WT mice with recombinant IL-24 (rIL-24) 1 h before administration with CCL_4_. Nonetheless, the levels of transaminases, percentages of hepatocyte death and expression of P-PERK and CHOP showed no statistical differences between vehicle and cytokine-treated mice (Supplementary Fig. 4A–C).

To obtain a closer insight into the intrinsic IL-24 function, we genetically depleted IL-24 in AML12 cells by using clustered regularly interspersed short palindromic repeats (CRISPR)/CRISPR associated protein 9 (Cas9) strategy and treated with Tm to mimic the pathological process in vivo. Cell Counting Kit-8 (CCK8) assays indicated that loss of intrinsic IL-24 impaired cell viability. Reciprocally, overexpression of IL-24 in AML12 benefited cell survival upon ER stress (Fig. 2f). Furthermore, annexin-V and propidium iodide staining showed that IL-24 attenuated late phase of apoptosis (Supplementary Fig. 5A, B). In addition, we detected the intracellular reactive oxygen species (ROS) level. Despite ROS accumulation over time, no difference was observed between vector and IL-24 KO groups (Supplementary Fig. 8A, B). Together, these results suggested that hepatocyte IL-24 plays a fundamental role in protecting ER stress-mediated cell death.

### Hepatocyte IL-24 deficiency activates PERK-eIF2α-CHOP pathway

**Fig. 3:**
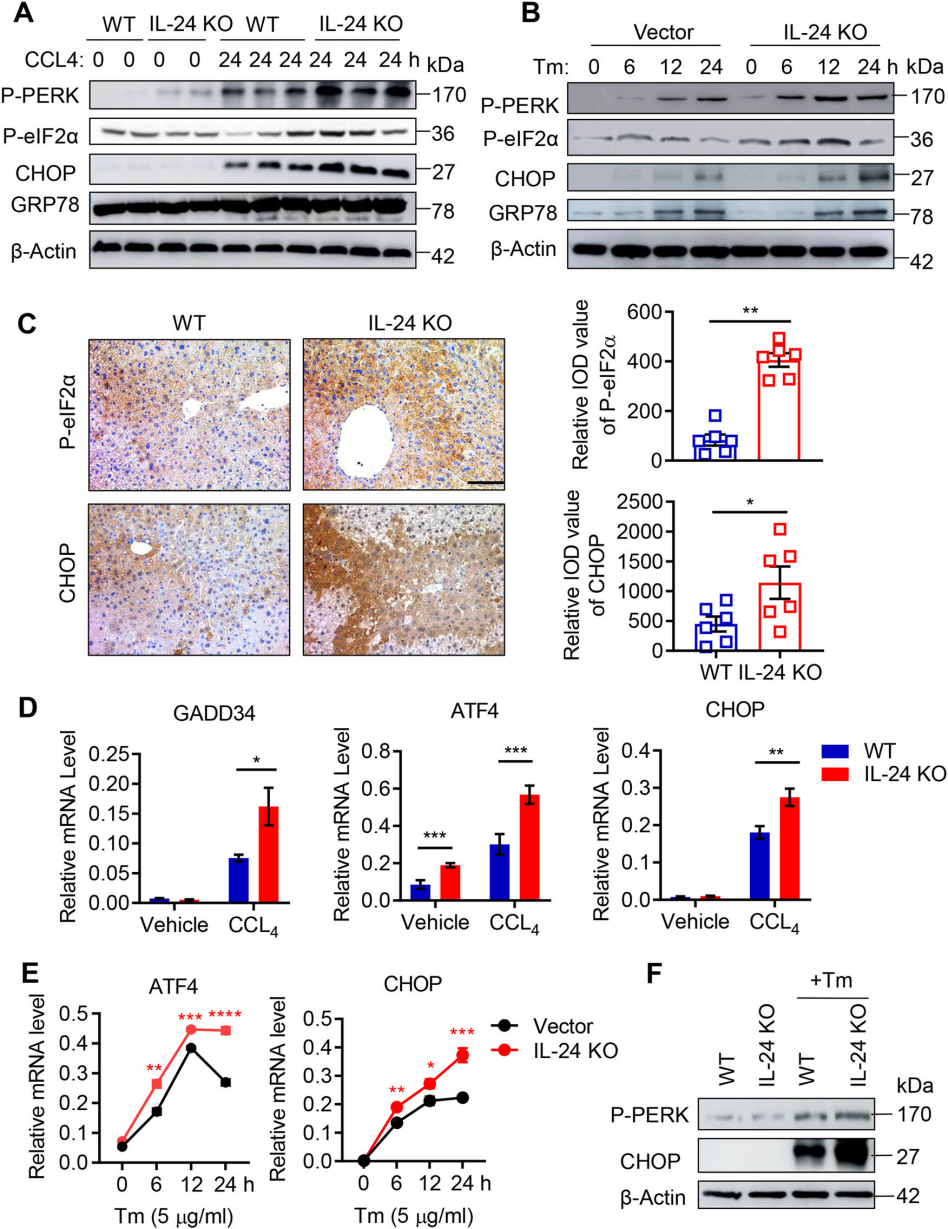
IL-24 deficiency facilitates PERK-eIF2α-CHOP UPR in hepatocyte. **a**, **b** P-PERK, P-eIF2α, CHOP and GRP78 protein levels in CCL_4_-treated WT and IL-24 KO mice, representatively (**a**). Tm-stimulated control and IL-24 KO AML12 cells (**b**), as evaluated by western blot at indicated time points. *n* *=* 3 independent experiments. **c** Representatively immunohistological staining of P-eIF2α and CHOP in the liver tissues from CCL_4_-treated WT and IL-24 KO mice. *n* *=* 5-8 mice. Scale bar, 100 µm. Results were represented in median integrated optical density (IOD) value. **d** GADD34, ATF4 and CHOP mRNA levels in the liver tissues from vehicle or CCL_4_-treated mice. *n* *=* 4-7 mice. **e** ATF4 and CHOP mRNA levels in indicated AML12 cells after Tm stimulation as assessed by qRT-PCR at indicated time points. **f** P-PERK and CHOP protein levels in Tm-stimulated primary hepatocytes from WT and IL-24 KO mouse representatively, as evaluated by western blot. *n* *=* 3 independent experiments. Data are presented as means ± SEM. *P < 0.05, **P < 0.01, ***P < 0.001, ****P *<* 0.0001. *P* values were determined by two-tailed *t* test.

To understand the mechanism behind hepatocyte stress, we assessed CHOP expression in both two acute liver injury models and Tm-exposed AML12 cells. Remarkably, loss of IL-24 in the mouse liver or hepatocytes unleashed CHOP expression, while introduction of IL-24 into AML12 efficiently diminished CHOP level (Fig. 3a, b and Supplementary Fig. 6A–C). In line with these findings, IL-24 depletion upregulated the expression of proapoptotic factors such as Bim and TRIB3, and yet downregulated antiapoptotic molecule Bcl2 (Supplementary Fig. 6D). To analyze how IL-24 was linked to ER stress, we further examined the UPR branches in the upstream of CHOP. Unexpectedly, either IRE1α phosphorylation or ATF6 expression appeared no difference between WT and IL-24 KO mice (Supplementary Fig. 6E). Noticeably, phosphorylation of PERK was selectively upregulated in the IL-24-deficient mouse liver or AML12 cells, and conversely downregulated upon IL-24 overexpression (Fig. 3a, b and Supplementary Fig. 6A). Accordingly, we evaluated the downstream molecules of PERK and found IL-24 deficiency robustly reinforced phosphorylation of eIF2α and expression of ATF4 and GADD34 in the stressed liver or AML12 cells, while overexpression of IL-24 in AML12 cells inhibited these molecules (Fig. 3a–e and Supplementary Fig. 6A–C). Immunohistochemical staining further confirmed higher levels of P-eIF2α and CHOP in IL-24-deficient liver (Fig. 3c). In aligned with these results, primary mouse hepatocytes isolated from IL-24-null mice manifested excessive activation of PERK-CHOP signal upon Tm-induced unresolved ER stress (Fig. 3f). Similarly, we also confirmed the activation of PERK-CHOP pathway in IL-24-deficient cells under in vitro CCL_4_ treatment (Supplementary Fig. 6F).

### Hepatocyte IL-24 selectively limits CHOP-mediated death signal

**Fig. 4:**
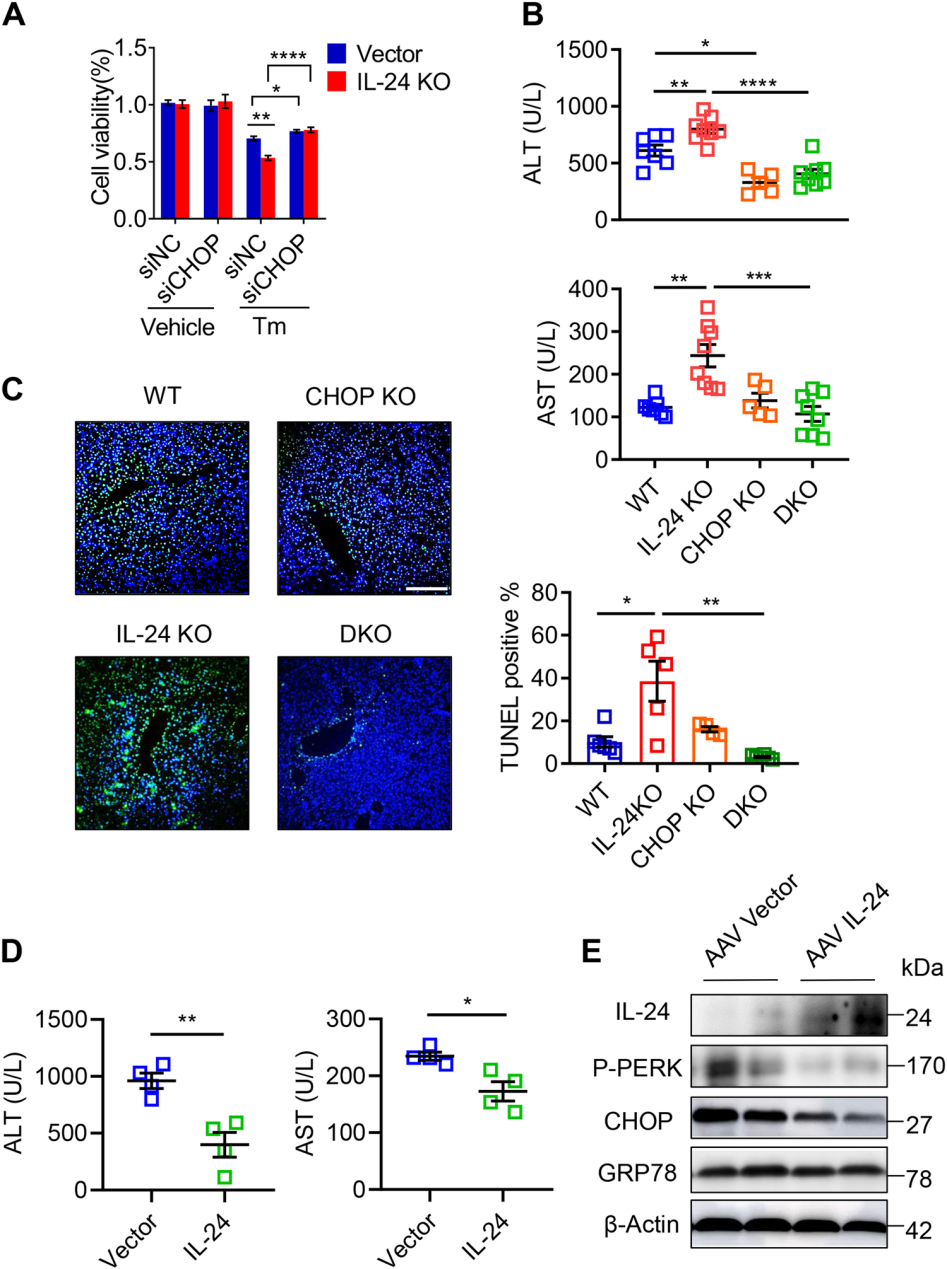
Hepatocyte IL-24 protects CHOP-executed cell death. **a** AML12 cells were treated with siCHOP or negative control (NC). Cell viability of indicated AML12 cells after Tm stimulation was assessed by CCK8 assay. *n* = 3 biological replicates. **b** Serum ALT (upper) and AST (lower) levels in WT, IL-24 KO, CHOP KO and IL-24/CHOP double KO (DKO) mice treated with CCL_4_ for 24 h. *n* *=* 5–8 mice. **c** Representatively TUNEL staining (left) of the liver tissues from CCL_4_-treated mice and quantification of TUNEL positive cells (right). Scale bar, 100 µm. **d** IL-24 KO mice were intravenously injected with AAV particles expressing an empty vector or mouse IL-24 8 weeks prior to CCL_4_ administration. Liver injury was assessed by serum ALT and AST levels. *n* *=* 4 mice. **e** Representatively IL-24, P-PERK, P-eIF2α, CHOP and GRP78 protein levels in the liver tissues from CCL_4_-exposed IL-24 KO mice with or without IL-24 re-expression. *n* *=* 3 independent experiments. Data are presented as means ± SEM. *P < 0.05, **P < 0.01, ***P < 0.001, ****P *<* 0.0001. *P* values were determined by two-tailed *t* test.

To explore whether CHOP is indispensable for IL-24 deficiency-related hepatocyte damage, we utilized siRNA targeting CHOP (siCHOP) to comprehend its executive role in ER-stressed hepatocytes. Administration of siCHOP to AML12 cells offset the marginal cell death caused by IL-24 depletion (Fig. 4a). In addition, we crossed IL-24-null mice with a CHOP-null strain to generate a double knockout (DKO) strain. In contrast to IL-24 KO counterparts, both CHOP KO and DKO mice rejected to CCL_4_-induced liver injury (Fig. 4b). As evident in histological staining, disruption of CHOP eliminated hepatocyte death in IL-24 KO mice (Fig. 4c). Therefore, these results suggest that IL-24 deficiency promotes hepatocyte death dependently on CHOP in the context of unresolved ER stress.

To better understand the intrahepatic function of IL-24, we treated the IL-24 null mice with IL-24-expressing adeno-associated viral (AAV) particles 8 weeks prior to CCL_4_ administration. As shown in Fig. 4d, e, re-expression of IL-24 in the liver markedly reduced the levels of serum transferases and P-PERK, P-eIF2α and CHOP. In addition, we checked the liver inflammation and detected lower IL1A and IL6 and higher TNFA mRNA expression in the IL-24-re-expression group (Supplementary Fig. 4F).

### Hepatocyte IL-24 attenuates liver damage by restricting the PERK-eIF2α branch

**Fig. 5:**
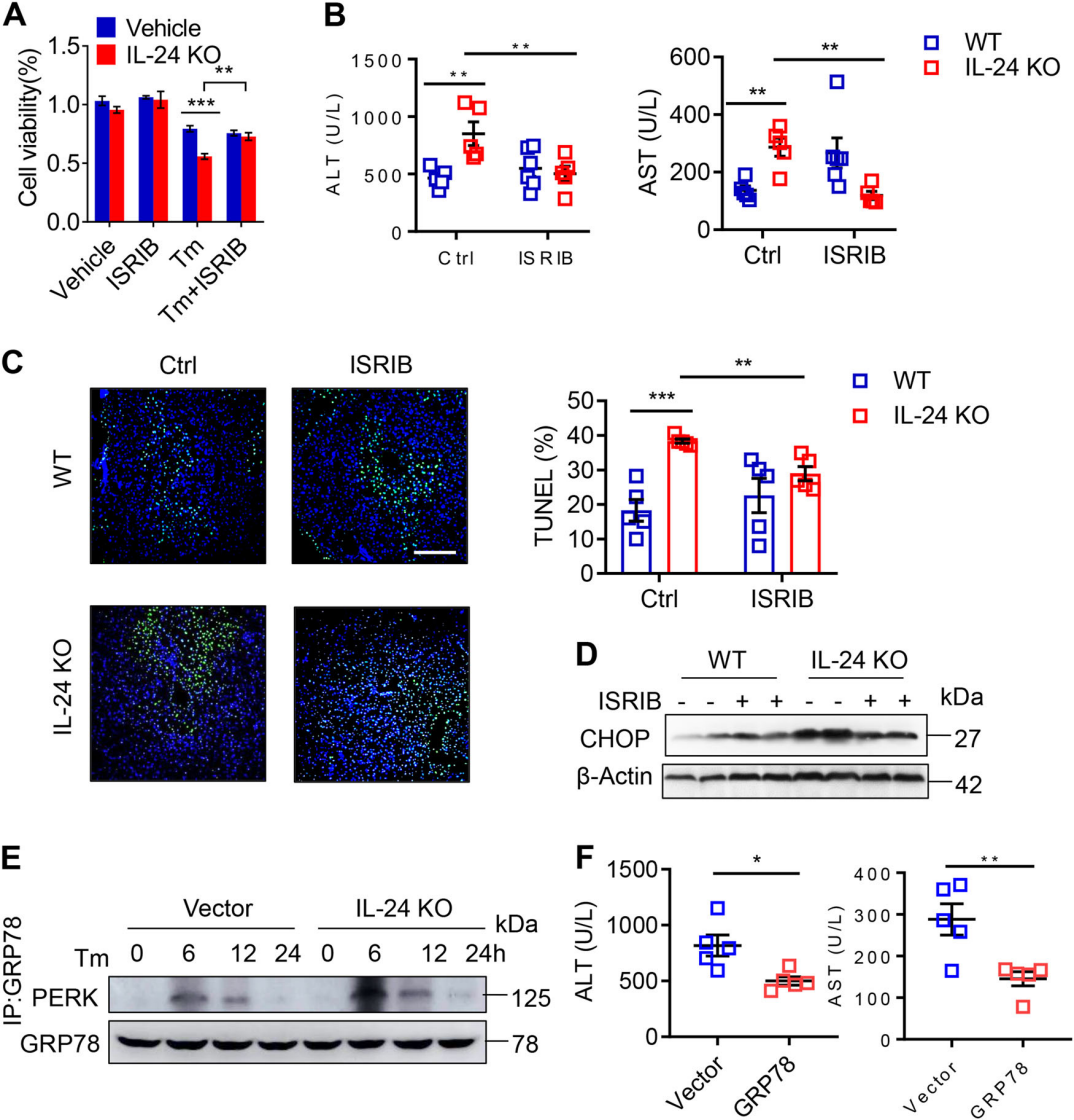
Perturbation of PERK-eIF2α signaling compensates ER homeostasis upon IL-24 loss. **a** Indicated AML12 cells were pre-treated with 200 nM ISRIB or DMSO (Vehicle) 24 h prior to Tm exposure. Cell viability of indicated AML12 cells after Tm stimulation was assessed by CCK8 assay. *n* = 3 biological replicates. **b**, **c** WT and IL-24 KO mice were intraperitoneally injected with vehicle or ISRIB (0.25 mg/kg) 60 min prior to CCL_4_ administration. Liver injury was assessed by serum ALT and AST levels (**b**) and representatively TUNEL positive cell ratios (**c**). *n* *=* 5 mice. Scale bar, 100 µm. **d** Representatively immunoblotting of CHOP in the liver tissues from CCL_4_-exposed mice with or without ISRIB treatment. *n* *=* 3 independent experiments. **e** Immunoblotting of PERK in the precipitates obtained by immunoprecipitation of endogenous GRP78 in indicated AML12 cells. *n* *=* 3 independent experiments. **f** IL-24 KO mice were intravenously injected with AAV particles expressing an empty vector or mouse GRP78 8 weeks prior to CCL_4_ administration. Liver injury was assessed by serum ALT and AST levels. *n* *=* 5 mice. Data are presented as means ± SEM. *P < 0.05, **P < 0.01, ***P < 0.001. *P* values were determined by two-tailed *t* test.

To ascertain the importance of PERK-eIF2α UPR branch, we built on an observation made with the inhibitor of the integrated stress response (ISRIB), which specifically blocks PERK-eIF2α signaling but unaffects ATF6 or inositol-requiring enzyme 1 (IRE1α) pathway^24^. Strikingly, pretreating AML12 with ISRIB compensated the viability loss for the lack of IL-24 but did not change the viability of WT cells (Fig. 5a). Next, we treated IL-24-null mice with vehicle or ISRIB 1 h prior to CCL_4_ administration. As indicated by serum aminotransferases, ISRIB reversed the deterioration of liver damage in IL-24-null mice but showed no profound impact on WT mice (Fig. 5b). H&E and TUNEL staining visualized an amelioration in hepatocyte death intensified by IL-24 depletion (Fig. 5c), which could be explained by the reduction of CHOP expression after ISRIB treatment (Fig. 5d).

It is known that the ER protein chaperon GRP78 binds to the cytoplasmic and ER luminal domains of PERK to prevent its activation^25^. Accordingly, we asked whether hepatocyte IL-24 affected the interaction between GRP78 and PERK. By performing immunoprecipitation, we pulled down GRP78 in AML12 cells and detected a significant binding of PERK after 6 h of Tm stimulation. In concert with the UPR degrees, its association with PERK was significantly enhanced in IL-24-deficient cells and was weakened in IL-24-overexpressed cells (Fig. 5e and Supplementary Fig. 7A). To obtain a functional relevance in vivo, we replenished chaperon expression in the mouse liver by intravenous injection of GRP78-expressing AAV. As shown in Fig. 5f and Supplementary Fig. 7B, C, overexpression of GRP78 strongly mitigated liver damage and PERK-CHOP activation in the CCL_4_-treated IL-24-null mice.

### Hepatocyte IL-24 predicts prognosis for patients with acute liver injury

**Fig. 6:**
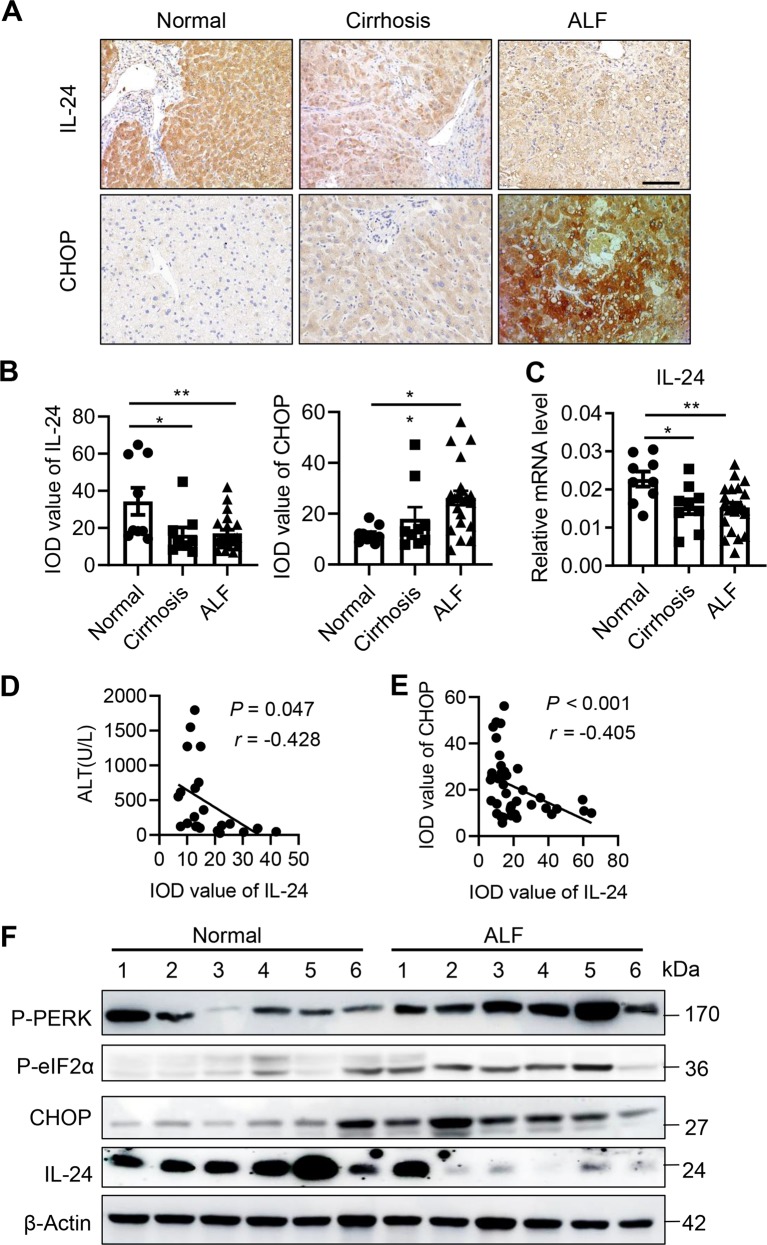
Hepatocyte IL-24 inversely correlates with liver function and CHOP expression in patients. **a** Representatively immunohistological staining of IL-24 and CHOP in the liver tissues from healthy donors (*n* = 9), cirrhosis (*n* = 9) and acute ALF (*n* = 22) patients. Scale bar, 100 µm. **b** Quantification of IL-24 protein levels in **a**, as represented in median integrated optical density (IOD) value. **c** IL-24 mRNA levels in the human liver tissues indicated in **a**, as assessed by qRT-PCR. Results are normalized to β-actin. *n* = 9–22 patients. Pearson correlation analysis between liver IL-24 protein level and serum ALT level (**d**) or liver CHOP protein level (**e**). *n* = 22. **f** Representatively immunoblotting of IL-24, P-eIF2α and CHOP in the liver tissues from healthy donors and acute ALF patients. *n* = 9 patients. Data are presented as means ± SEM. *P < 0.05, **P < 0.01. *P* values were determined by two-tailed *t* test.

**Fig. 7:**
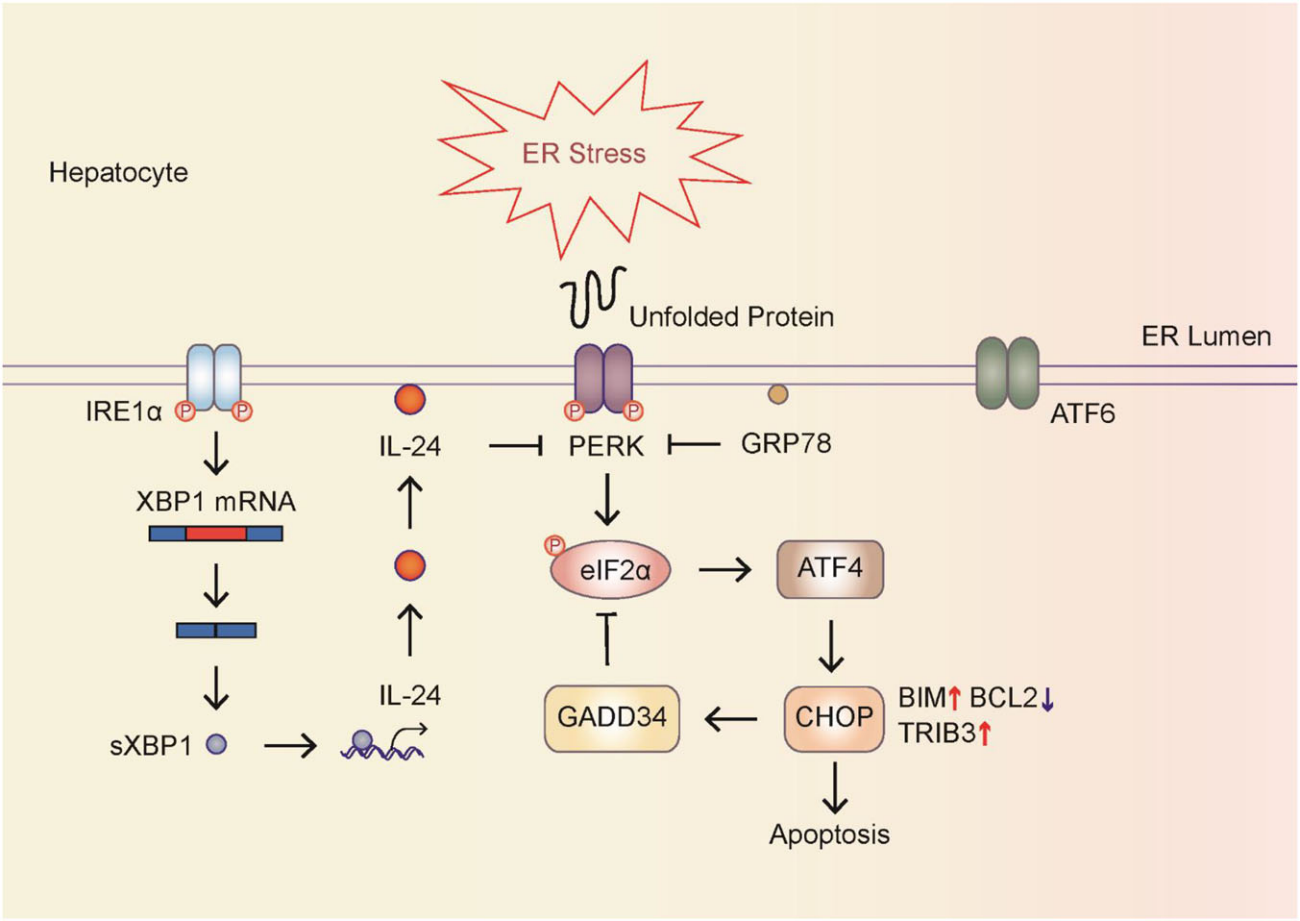
Schematic model showing the interaction between cytoplasmic IL-24 and UPR modulators within hepatocytes. Hepatocyte ER stress engages sXBP1 for upregulating IL-24 transcription, which in turn improves ER homeostasis and represses CHOP-mediated cell death by harnessing PERK-eIF2α branch reaction.

To examine the biological significance of IL-24 in clinical situations, we collected liver tissue and serum samples from 9 healthy donors, 9 patients with liver cirrhosis and 22 patients with acute liver failure (ALF). Serum IL-24 levels in both two groups of liver injury patients were as low as that in heathy donors (data not shown), which was aligned with what we found in CCL_4_-induced liver injury mouse models. Nonetheless, immunohistochemistry, immunoblots and qRT-PCR indicated that IL-24 expression was reduced in patients with liver cirrhosis and even lower in those with ALF (Figs. 6a–c, f and 7), whereas CHOP expression was concomitantly escalated in cirrhosis and ALF patients (Fig. 6a, f), suggestive of a strong correlation between IL-24 expression and hepatocyte stress. In a further analysis of ALF patients, we obtained the individual liver function test before liver transplant and found IL-24 expression in hepatocytes was negatively related to serum ALT level as well as liver CHOP expression (Fig. 6d, e). Taken together, hepatocyte IL-24 may function as a prognosis predictor for patients with acute liver injury.

## Discussion

UPR is executed through three ER transmembrane stress sensors: IRE1α, PERK and activating transcription factor 6 (ATF6)^5^. Activated IRE1α splices XBP1 mRNA, which encodes transcription factor to increase the protein-folding capacity and degrade misfolded proteins. While IRE1α engages STAT3 pathway to promote liver regeneration upon liver injury^26^, XBP1 switches pro-survival to proapoptotic signal cascades through multiple gene regulation^27,28^. It has been reported that ATF6 exerts a pro-inflammatory effect on ischemia-reperfusion liver injury^29^. PERK antagonizes UPR by reducing the flux of protein translocating and phosphorylating eIF2α, a pervasive translation initiation factor, which inhibits ribosome assembly and translation. However, eIF2α selectively upregulates the transcription factor ATF4 and its downstream target CHOP. Sustained ER stress engages CHOP to enhance UPR and inflammation signaling and lead to apoptosis^22,30^ (Fig. 7). The mouse models in our study manifested activation of three UPR sensors and CHOP. The latter was also found to be upregulated in human cirrhosis and ALF patients. Given the recovery of liver function in most cases, we hypothesized an unknown machinery restoring hepatocyte ER homeostasis.

A recent review has summarized the functions of IL-20 family cytokines in liver diseases^31^. IL-24 was suggested to function as a protective cytokine during liver inflammation through binding to its corresponding receptors. In the present study, however, administration of recombinant IL-24 did not ameliorate CCL_4_-induced liver damage. Noticeably, we detected abundant IL-24 expression in the mouse or human liver under normal condition. Since exogenous IL-24 in melanoma cells cause ER stress through a secretion-independent manner^19^, we asked whether the endogenous IL-24 in hepatocytes interacts with ER components under normal or ER-stressed conditions. In the CCL_4_ model, hepatocyte IL-24 increases instantaneously and then returns to baseline as the liver function recovers. Nonetheless, serum IL-24 was undetectable in CCL_4_-treated mice and human cirrhosis and ALF patients, excluding its engagement as a “hepatokine” in UPR condition. Importantly, targeting ER stress-related transcription factors (ATF4, ATF6, XBP1 and CHOP) significantly reduced the mRNA level of hepatocyte IL-24. However, only silencing of XBP1 deprived IL-24 expression under either normal or deleterious circumstance. As anticipated, sXBP1 directly binds to *Il24* promoter and reinforces its transcription. This reflects a physiological function of hepatic XBP1 and also raises a possibility that intrinsic IL-24 may regulate stress signals.

Since adenovirus-mediated overexpression causes protein synthesis overload and induces potential UPR, we employed gene knockout mice to improve our understanding of cytosolic IL-24 in ER stress and liver damage. Remarkably, we found IL-24-null mice were more sensitive to CCL_4_-induced liver injury than WT counterparts. While either ATF6 expression or IRE1α phosphorylation was unaffected, P-PERK, P-eIF2α, CHOP and GADD34 exhibited excessive expression in the IL-24-deficient mouse liver. In addition, our results showed elevated levels of Bim and TRIB3 and a reduced level of Bcl2 in IL-24-null mice, which might be a consequence of CHOP activation^3,11^. Effects of hepatocyte IL-24 on PERK-eIF2α-CHOP branch and cell death were corroborated by depleting or introducing IL-24 in AML12 cells. Furthermore, knocking out CHOP in IL-24-null mice or knocking down CHOP in IL-24-null AML12 protected ER stress-associated hepatocyte damage. It has been reported that eIF2α phosphorylation acts as a central event sensitizing stressed cells to death^12,27,29^. Accordingly, we found that the extensive liver damage in IL-24-null mice could be alleviated by administration of ISRIB, which selectively reverses the effects of eIF2α phosphorylation^24^. Clinically, we observed a concomitant downregulation of IL-24 and upregulation of CHOP in the cirrhosis and ALF tissues as compared with the healthy liver. Taken together, these findings demonstrated the protective role of IL-24 in resolving hepatocyte ER stress is implemented by perturbation of PERK-eIF2α-CHOP pathway. However, other downstream targets of IL-24 remain to be investigated.

As one of the most important ER chaperons, GRP78 is essential in conjunction with misfolded proteins and is important for maintaining ER homeostasis^8,32^. Given the reinforced ER stress in IL-24-null hepatocytes, GRP78 conserved its affinity to combine and sequester overreacted PERK. Despite this, redundant PERK phosphorylated itself and triggered the downstream signaling. Previous study indicated that in vivo overexpression of GRP78 using an adenovirus vector could attenuate ER stress-associated liver steatosis^33^. In this study, introduction of GRP78 in the IL-24-deficient mouse liver by AAV infection attenuated PERK-facilitated hepatocyte stress. This may provide a potential therapeutic opportunity for UPR-related human liver diseases, especially those with low hepatocyte IL-24 expression.

To our knowledge, a variety of cytokines, including those expressed or secreted by hepatocytes, evoke inflammatory responses and promote cell death in liver diseases. In a diet-induced steatohepatitis mouse model, hepatocyte IL-1α was found to be upregulated in response to ER stress, which in turn enhanced CHOP expression; IL-1α released from necrotic hepatocytes accelerates steatohepatitis via induction of inflammatory cytokines^34,35^. In lipopolysaccharide (LPS)-induced liver injury, hepatocyte-derived IL-7 augmented CD8+T cell cytotoxic activity and promoted the development of autoimmune diseases^36^. In the present study, we showed that intracellular IL-24 uniquely benefited hepatocyte ER homeostasis, exerting an anti-inflammatory effect. However, it has not been defined whether hepatocyte can secrete IL-24 under pathological conditions and how it affects hepatocytes or immune cell populations in an autocrine or paracrine fashion.

Conclusively, we uncovered that cytosolic IL-24 is critical for protecting ER stressed hepatocytes from death, which may be a good diagnostic and therapeutic target for clinical liver diseases.

## Materials and methods

### Patients

Cirrhosis and liver failure tissues were collected from liver transplant recipients treated in Department of Liver Surgery, Renji Hospital, School of Medicine, Shanghai Jiaotong University. Normal liver tissues were collected from the healthy transplant donors through liver biopsy. Eligibility Criteria: All patients were aged ≥20 years. Liver cirrhosis criteria include: based on the patients’ laboratory tests and the findings from imaging studies, (a) ultrasonography, computed tomography and pathological evidence that revealed liver cirrhosis, splenomegaly, esophageal varices, and/or ascites; and (b) no hepatic encephalopathy, hepatocellular carcinoma or low estimated glomerular filtration rate (eGFR; <15 mL/min/1.73 m^2^), or acute renal failure^37^. ALF criteria include: (a) hepatic encephalopathy of any degree; (b) evidence of moderately severe coagulopathy (international normalized ratio (INR) greater than or equal to 1.5); (c) presumed acute illness within 26 weeks of onset of symptoms; and (d) no pathologically cirrhosis^38^. All samples were collected randomly with informed consent, and the experiments were approved by the ethical review committee of the World Health Organization Collaborating Center for Research in Human Production (authorized by the Shanghai Municipal Government).

### Mice

All mice used in this study were in C57BL/6J background, collected randomly and no blinding was done. IL-24 knockout (KO) mice were generated in ShanghaiTech University. The targeted Embryonic stem (ES) cells were ordered from The Knockout Mouse Project (KOMP) Repository, in which the insertion of Velocigene cassette ZEN-Ub1 created a deletion of size 3306 bp between positions 132779010-132782315 of Chromosome 1 (Genome Build37). These ES cells were injected into albino C57BL/6J blastocysts and the following inbred strain was generated by backcrossing breeding. The following PCR primers were used to identify WT (571 bp) and KO (338 bp) alleles: 5′- GTACCCACTCCAATGCATACATT -3′, 5′- GCTCATCCAGGATGAAGCTACAC -3′, and 5′-GAAACCAGGCAAATCTCCACTCC -3′. The CHOP KO mice and Alb^Cre^ transgenic mice were purchased from Jackson Laboratories. We crossed the CHOP KO and IL-24 KO strains to produce the double knockout (DKO) strain. The Xbp1^F/F^ strain, a.pngt from Dana Farber Cancer Institute, USA, was previously described^39^. Mice devoid of XBP1 selectively in hepatocytes were generated by breeding the Xbp1^F/F^ mice with Alb^Cre^ strain. For acute liver injury model, CCL_4_ dissolved in olive oil was injected intraperitoneally into 8-week-old female mice at a dose of 2 ml/kg^26^. Besides, mice were fasted overnight and administered 500 mg/kg APAP (Sigma) by oral gavage^12^. In some settings, mice were intraperitoneally injected with 0.25 mg/kg ISRIB (Selleck) or recombinant IL-24 (R&D) at a dose of 5 μg per mouse 1 h prior to CCL_4_ administration. All mice were maintained under specific pathogen-free (SPF) conditions, on a 12 h light-dark cycle. All mouse experiments were approved by the Shanghai Administrative Committee for Laboratory Animals.

### Statistical analysis

At least three biological replicates were used in each experiment unless otherwise stated. Data were analyzed with GraphPad Prism 7 and were presented as the mean ± standard error of the mean (SEM). Two-tailed Student’s *t* tests were performed to assess the statistical significance of differences between groups. Pearson correlation coefficients (*r*) were calculated to assess correlation and statistical significance was assessed by a two-tailed *t* test of *r* = 0.

### Supplementary methods

More detailed methods including Isolation of primary mouse hepatocytes and cell cultures, siRNA, sgRNA and gene transfection, Recombinant AAV construction and In vivo transduction, Immunohistochemistry, Immunofluorescence, Immunoprecipitation, Western blot, Quantitative PCR, dual luciferase assay, chromatin immunoprecipitation, apoptosis and ROS analysis are provided in supplementary information.

## Supplementary information

Reproducibility Checklist

Supplementary Methods

Supplementary Figure 1

Supplementary Figure 2

Supplementary Figure 3

Supplementary Figure 4

Supplementary Figure 5

Supplementary Figure 6

Supplementary Figure 7

Supplementary Figure 8

Supplementary Figure 9

Supplementary Figure 9

Supplementary Figure Legends

Detailed Attribution of Authorship
