# Supplementary material for: Intracellular XBP1-IL-24 axis dismantles cytotoxic unfolded protein response in the liver
Source: Cell Death Dis. 2020 Jan 6;11(1):17. doi: 10.1038/s41419-019-2209-6 (PMC6944701; doi:10.1038/s41419-019-2209-6)
Supplement: Supplementary file 2 — Supplementary Methods [file 41419_2019_2209_MOESM2_ESM.docx]

**Isolation of primary mouse hepatocytes and cell cultures.** The mouse liver was perfused with an EGTA-buffer (37°C) at a constant flow of 5 ml/min for 8 minutes via the hepatic portal vein. Next, a secondary perfusion with a solution of collagenase I (Sigma-Aldrich) for 10 minutes (2 ml/min) is required for a completely digestion. The liver was disrupted gently to release hepatocytes into a suspension buffer. Subsequently, the liver capsule was filtered through a 70-µm cell strainer and the primary hepatocytes were collected after three cycles of centrifugations at 400 rpm for 5 min at 4°C. A suspension of 1×10^6^ cells/mL was successively seeded in culture plates. Murine hepatocyte cell line AML12 was from The First Affiliated Hospital of Nanjing Medical University and has been authenticated for its origin of mouse hepatocytes through STR profiling. Primary hepatocytes and AML12 cells were cultured in William's E Medium (Gibco) supplemented with 1X insulin-transferrin-selenium supplement (Gibco), 1X sodium pyruvate (Gibco), 40 ng/ml dexamethasone (Sigma-Aldrich) and 10% FBS (Gibco), incubated at 37 °C and 5% CO_2_, and were tested for mycoplasma contamination once every three months. In some settings, cells were treated with 5 μg/ml Tm (Sangon Biotech) or 200 nM ISRIB (Selleck).

**siRNA, sgRNA and gene transfection.** The siRNAs were transiently transfected into AML12 cells by using Lipofectamine RNAiMAX (Invitrogen) following manufacturer’s instructions. A non-target siRNA was used as negative control. The siRNA sequences are listed as below: mouse XBP1, CCAAGCUGGAAGCCAUUAATT; mouse CHOP, CCAGAUUCCAGUCAGAGUUTT; mouse ATF4, CUCCCAGAAAGUUUAAUAATT; mouse ATF6, GCAGUCGAUUAUCAGCAUATT. Stable knockout of IL-24 in AML12 cells was generated by lentiviral-based delivery of sgRNA/cas9 components. Briefly, sgRNA targeting the exonic region of murine *Il24* gene (5′-GAAGGATTAGGCTCAGGCAG-3′) were subcloned into the lentiviral vector GV393 (U6-sgRNA-EF1a-Cas9-FLAG-P2A-EGFP) (Genechem, China), while a non-target sgRNA (5′-CGCTTCCGCGGCCCGTTCAA-3′) was used as a negative control. IL-24 (NM_053095)- and sXBP1 (NM_001271730)- overexpression construct was generated by subcloning PCR-amplified full-length cDNA into a GV358 (Ubi-MCS-Flag-SV40-EGFP-IRES-puromycin) lentiviral vector (Shanghai Genechem). An empty vector was used as a negative control. Viral particles were packaged in 293T cell and used to infect AML12 cells in the presence of 8 µg/ml polybrene followed by puromycin selection.

**Recombinant AAV construction and In vivo transduction.** GRP78-overexpression construct was generated by subcloning PCR-amplified full-length *Il24* (NM_053095) or *Hspa5* (NM_022310) cDNA into a GV461 (CMV-betaGlobin-MCS-SV40 PolyA) AAV vector (Shanghai Genechem). An empty vector was used as a negative control. IL-24 KO mice of 5 weeks were intravenously injected with 2 x 10^11^ vector-genome (vg) AAV 8 weeks prior to CCL_4_ administration.

**Immunohistochemistry and Immunofluorescence.** Immunohistochemistry for target molecules was performed on serial sections from human or mouse liver tissues. Sections were deparaffinized, subjected to antigen retrieval, and incubated with primary antibodies against IL-24 (Abcam, ab115207), P-eIF2a (Huabio, ET1603-14) and CHOP (Huabio, ET1703-05). All responses were followed by staining with the corresponding HRP-conjugated secondary antibody (Jackson Immuno Research Laboratories). The stained slides were assessed with integrated optical density (IOD) using ImageJ software. The apoptotic cells were defined by using In Situ Cell Death Detection Kit (Roche) following manufacturer’s protocol and quantified by calculating positively stained cells in at least five randomly chosen HPFs of each slide.

**Immunoprecipitation.** Cell samples were collected and lysed in IP lysis buffer (Thermo Fisher) containing protease inhibitor cocktail (Merck Millipore) for 30 minutes. After an insoluble product-clear step by full speed centrifuge, the supernatant was harvest and incubated with anti-GRP78 (Abcam, ab21685) antibody and protein A beads (Thermo Fisher) at 4 °C overnight. The beads were collected and washed extensively, and the immuno-complex was eluted with western blot loading buffer.

**Western blot.** Cell or tissue lysates were separated on 6-8% polyacrylamide-SDS gels and transferred to a nitrocellulose membrane using transfer buffer (25 mM Tris, 192 mM glycine and 10% methanol). The blots were blocked with 5% non-fat milk in PBS containing 0.05% Tween-20 for 1 h and then probed overnight at 4°C in PBST with primary antibodies. Next, the blots were incubated with a secondary antibody conjugated to horseradish peroxidase (HRP) (1:5,000, Jackson Immuno Research Laboratories) and detected with a ChemiDoc XRS system (Bio-Rad). Primary antibodies used in western blot are listed below: anti-Flag (14793S), anti-P-PERK (3179S), anti-ATF4 (11815S) and anti-CHOP (2895S) were from Cell Signal Technology, anti-human IL-24 (ab115207), anti-P-eIF2a (ab32157) and anti-GRP78 (ab21685) were from Abcam, anti-mouse IL-24 (MAB2786) was from R&D Systems, anti-PERK (sc-377400) was from Santa Cruz.

**Quantitative PCR (qPCR).** Total RNA samples used for RT-qPCR were isolated by using an RNeasy kit (ΒioTeke) with an additional on-column DNase-I digestion step. Total RNA or purified mRNA was reverse transcribed with PrimeScript™ RT Master Mix (Takara) using Oligo dT primers to obtain complementary DNA. qPCR was carried out by using SYBR Premix Ex Taq II (Takara). β-actin was used as an internal control. The primers used in this study are: mouse GRP78(F:5’- TCATCGGACGCACTTGGAA -3’;R:5’- CAACCACCTTGAATGGCAAGA -3’); mouse CHOP(F:5’- CTGGAAGCCTGGTATGAGGAT-3’;R:5’- CAGGGTCAAGAGTAGTGAAGGT -3’) ; mouse ATF4(F:5’ - CTCTTGACCACGTTGGATGAC - 3’; R: 5’ - CAACTTCACTGCCTAGCTCTAAA -3’) ; mouse IL-24 (F:5’- GAGCCTGCCCAACTTTTTGTG -3’;R:5’- TGTAGTCCCCAACTCATCTGTG -3’); mouse sXBP1 (F: 5’- CTGAGTCCGAATCAGGTGCAG -3’; R: 5’- GTCCATGGGAAGATGTTCTGG - 3’); mouse ATF6 (F: 5’- TCGCCTTTTAGTCCGGTTCTT -3’; R: 5’- GGCTCCATAGGTCTGACTCC - 3’); mouse GADD34 (F: 5’- GAGGGACGCCCACAACTTC -3’; R: 5’- TTACCAGAGACAGGGGTAGGT - 3’); mouse IL6 (F: 5’- TAGTCCTTCCTACCCCAATTTCC -3’; R: 5’- TTGGTCCTTAGCCACTCCTTC - 3’); mouse IL1A (F: 5’- CGAAGACTACAGTTCTGCCATT -3’; R: 5’- GACGTTTCAGAGGTTCTCAGAG - 3’); mouse Bim (F: 5’- GACAGAACCGCAAGGTAATCC -3’; R: 5’- ACTTGTCACAACTCATGGGTG - 3’); mouse TRIB3 (F: 5’- GCAAAGCGGCTGATGTCTG -3’; R: 5’-AGAGTCGTGGAATGGGTATCTG - 3’); mouse Bcl2 (F: 5’- ATGCCTTTGTGGAACTATATGGC -3’; R: 5’- GGTATGCACCCAGAGTGATGC - 3’); mouse TNFA (F: 5’- CTCTTCTGTCTACTGAACTTC -3’; R: 5’- CTCCTGGTATGAGATAGCAA - 3’); mouse β-actin (F:5’- ACCCACACTGTGCCCATCTAC -3’;R:5’- AGCCAAGTCCAGACGCAGG -3’); human IL-24(F:5’- CACACAGGCGGTTTCTGCTAT-3’; R:5’- TCCAACTGTTTGAATGCTCTCC -3’); human β-actin (F: 5’- GGGAAATCGTGCGTGACATTAAG -3’; R: 5’- TGTGTTGGCGTACAGGTCTTTG - 3’).

**Dual luciferase assay.** A DNA fragment of *Il24* (-1036 ~ -598 bp in the upstream of transcription start site) was subcloned into a luciferase reporter vector pGL4 (Promega). AML12 cells were cultured in 24-well plates for 24 hours, then transfected with siRNAs. The cells were co-transfected with luciferase reporter plasmid and renilla luciferase plasmid (an internal control) at a ratio of 10:1. Twenty-four hours later, cells were treated with Tm (5 μg/ml). Cells from each independent well were harvested and detected by Dual-Luciferase Reporter Assay System (Promega) at indicated time. Get the relative light unit (RLU) by normalizing to renilla luminescence activities.

**Chromatin immunoprecipitation (ChIP).** ChIP was performed by using Chromatin Immunoprecipitation kit (Thermo Fisher). In brief, AML12 cells were cross-linked with 1% final formaldehyde containing 1X protease inhibitor cocktail. Then the chromatin was collected for sonication and incubated with 4μl of antibody (Anti-Flag, Cell Signal Technology) per reaction. After being mixed with magnetic beads for 2 hours, the beads were washed 3 times and the DNA was extracted and precipitated. The purified DNA template was analyzed by conventional PCR using primers specific for target gene promoter. The information for primers is as follows:

*Il24*-ChIP-F (sXBP1): 5’ - CACAGCAAGTGCTAAATGAAGCT- 3’

*Il24*-ChIP-R (sXBP1): 5’ - CTGTATCTCCAGCTAGCAAGGTG - 3’.

**Apoptosis and ROS analysis.** AML12 cells were stimulated by Tm, and 1~5x10^5^ cells were collected by centrifugation. The cells were resuspended with the 1x Binding Buffer and incubated with 5μL FITC-conjugated annexin V Annexin FITC (BD Biosciences, USA) and 5μL PI (BD Biosciences, USA) or 5μL ROS Fluorescent Probe-DHE (Vigorous Biotechnology, CN) respectively to each tube according to the experimental protocol. Then samples were analyzed by fluorescence-activated cell sorter (FACS).
