# Supplementary figures and images for: Intracellular XBP1-IL-24 axis dismantles cytotoxic unfolded protein response in the liver
Source: Cell Death Dis. 2020 Jan 6;11(1):17. doi: 10.1038/s41419-019-2209-6 (PMC6944701; doi:10.1038/s41419-019-2209-6)

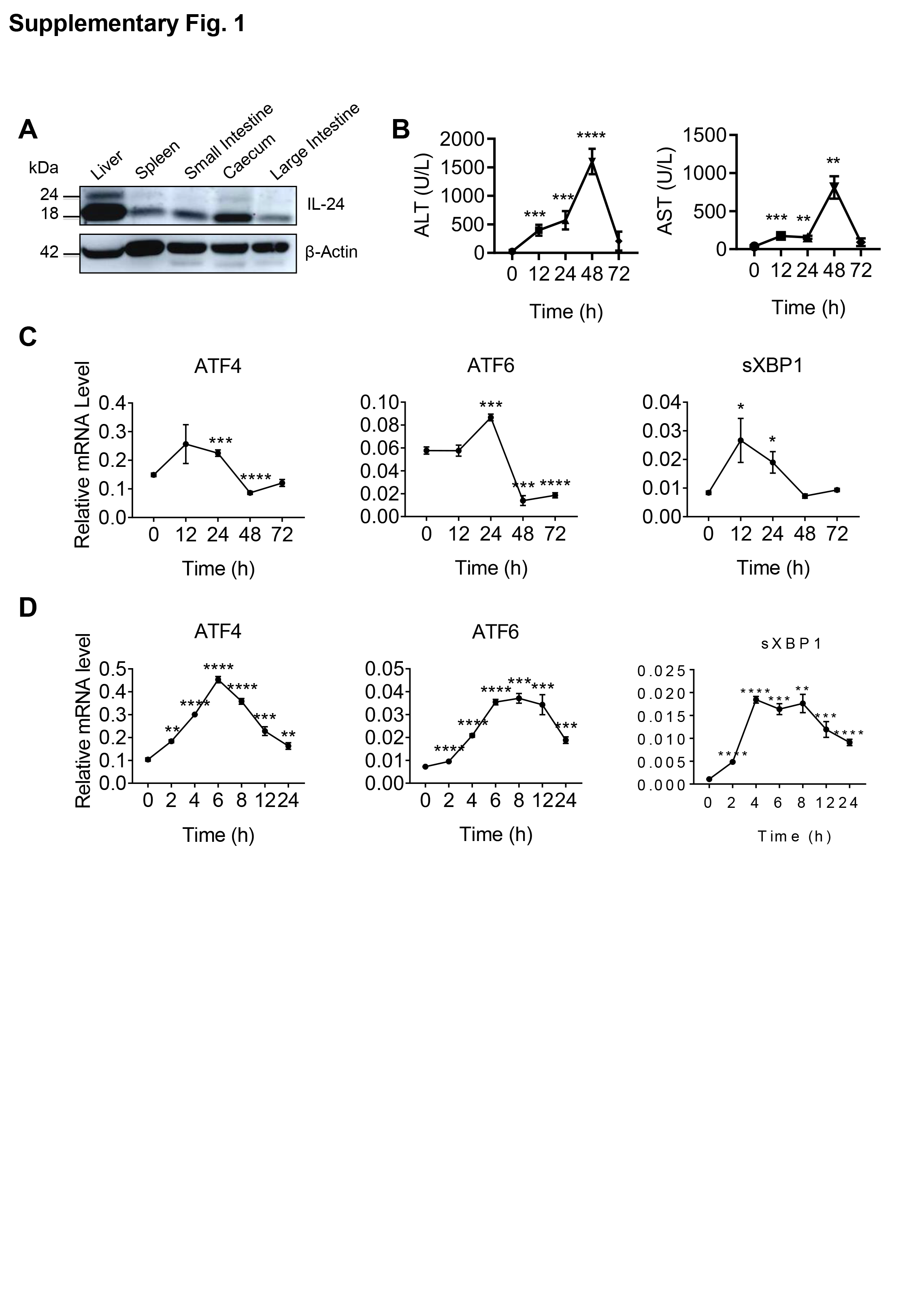

Supplement: Supplementary file 3 — Supplementary Figure 1 [file 41419_2019_2209_MOESM3_ESM.png]

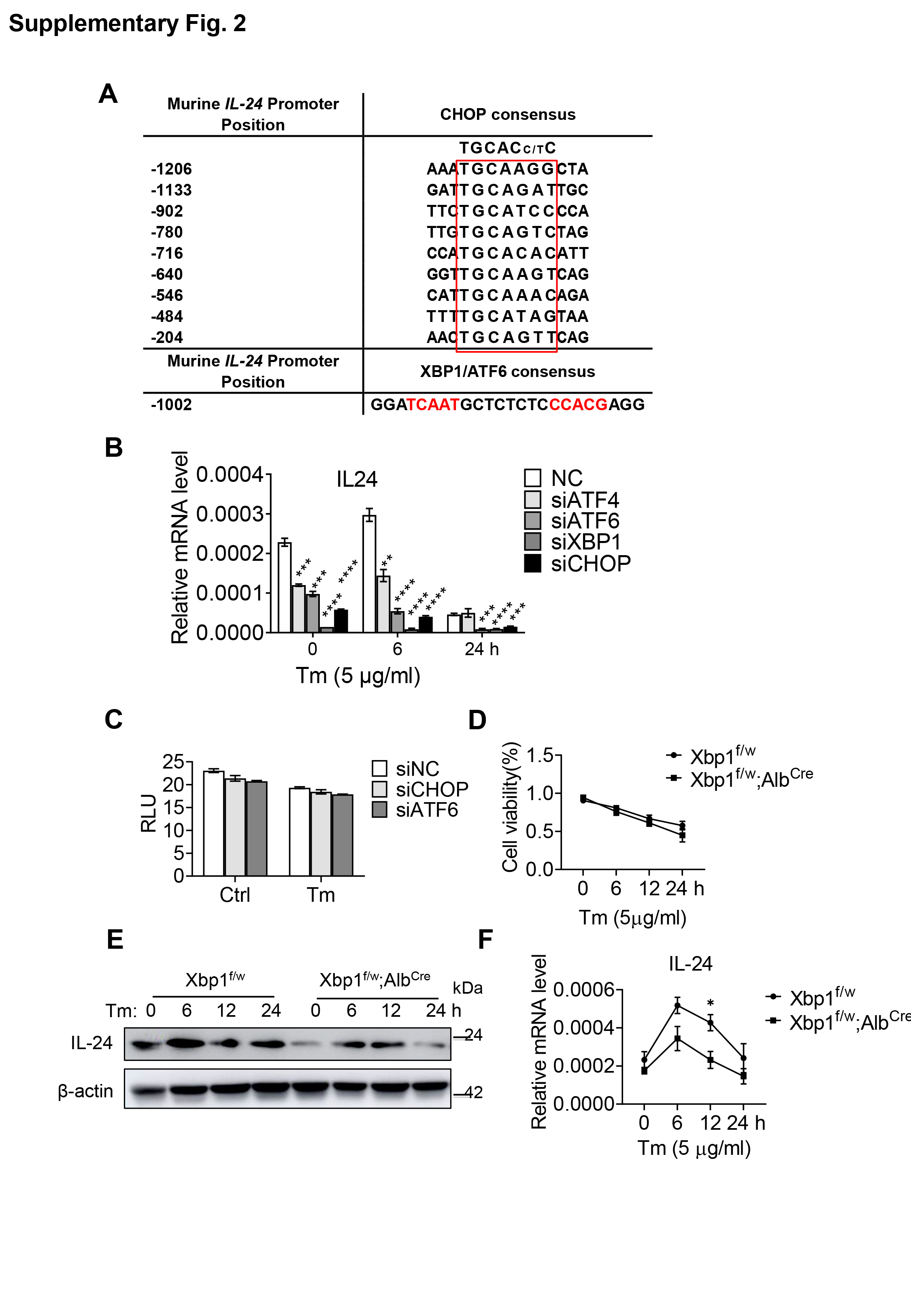

Supplement: Supplementary file 4 — Supplementary Figure 2 [file 41419_2019_2209_MOESM4_ESM.png]

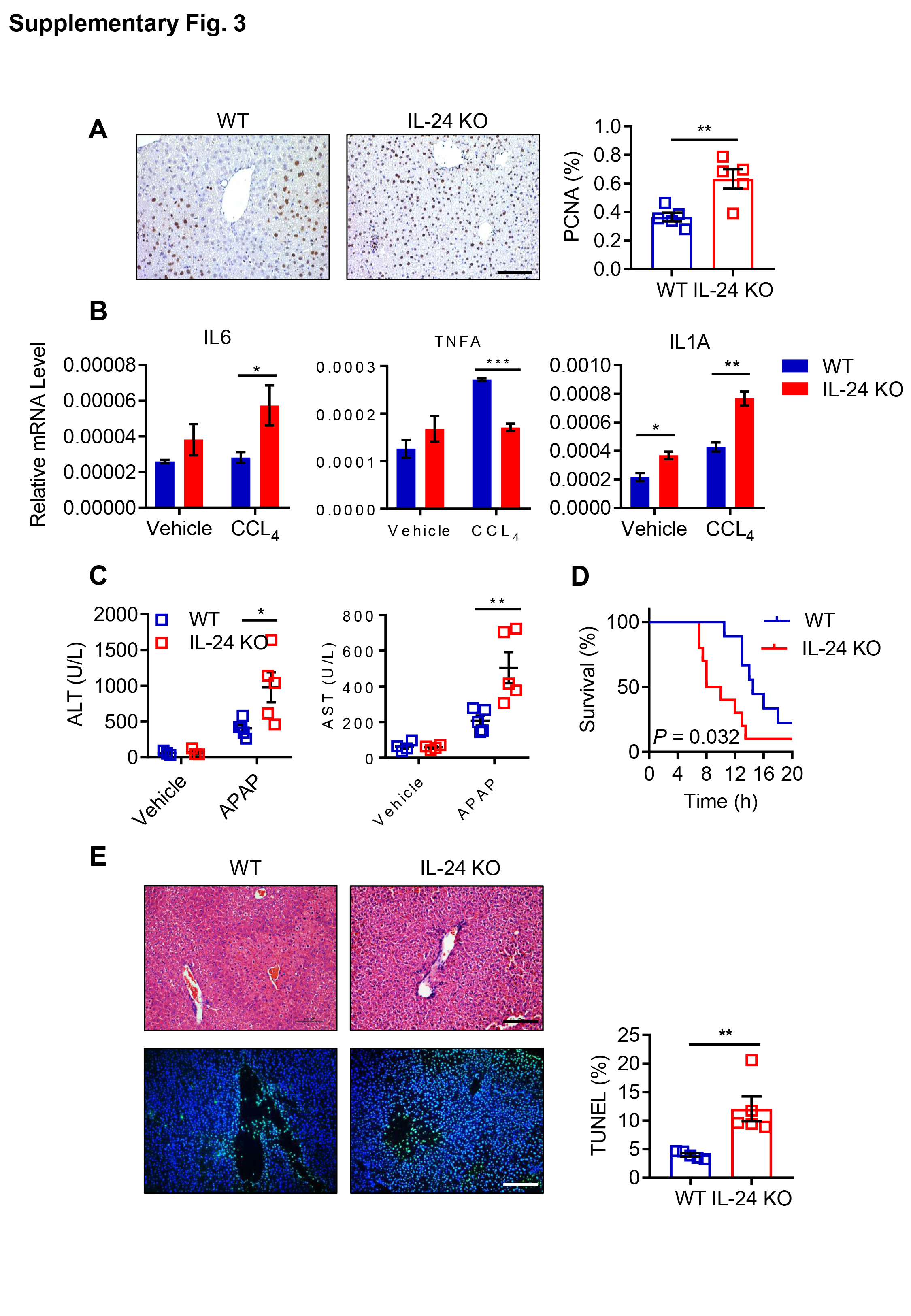

Supplement: Supplementary file 5 — Supplementary Figure 3 [file 41419_2019_2209_MOESM5_ESM.png]

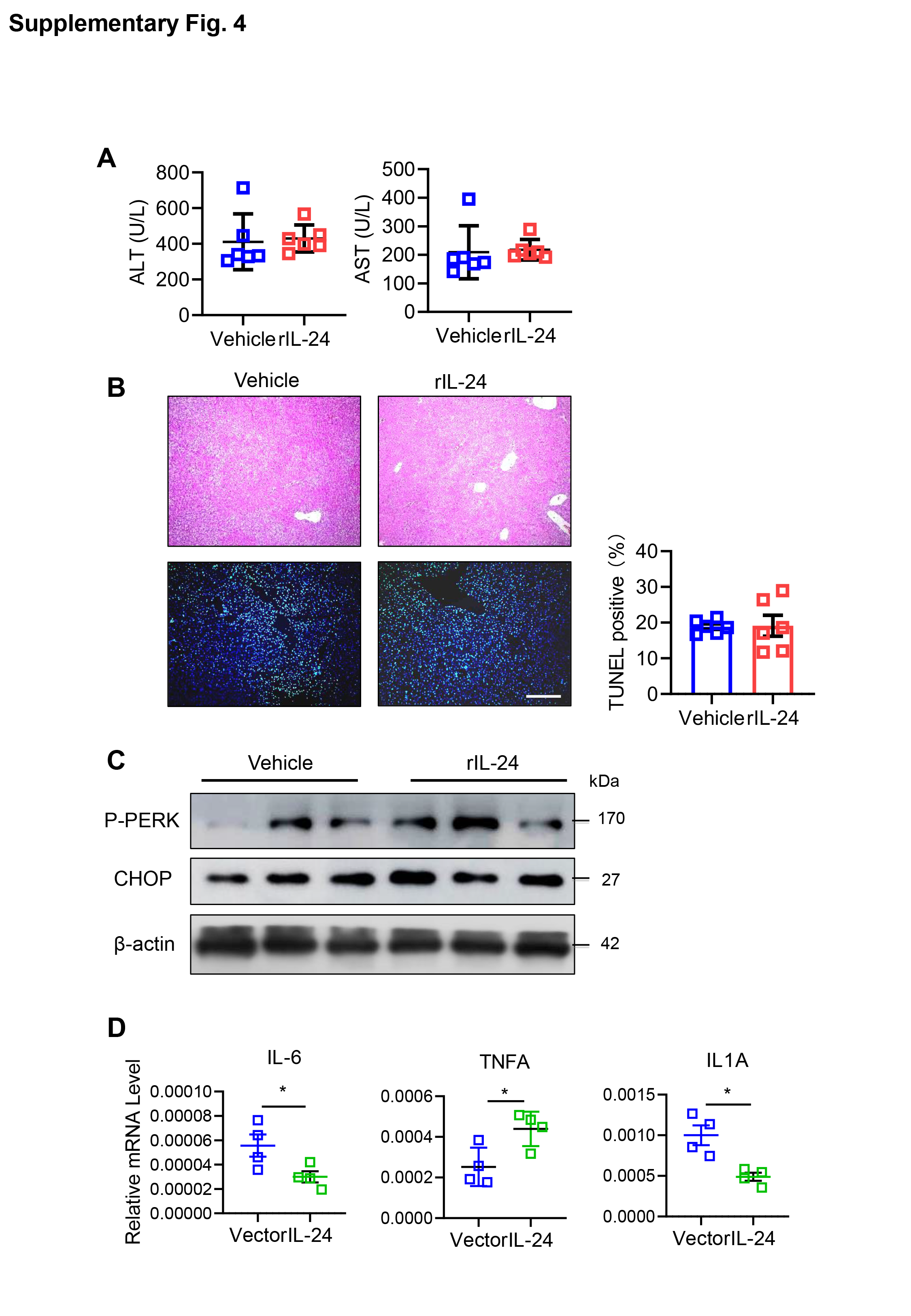

Supplement: Supplementary file 6 — Supplementary Figure 4 [file 41419_2019_2209_MOESM6_ESM.png]

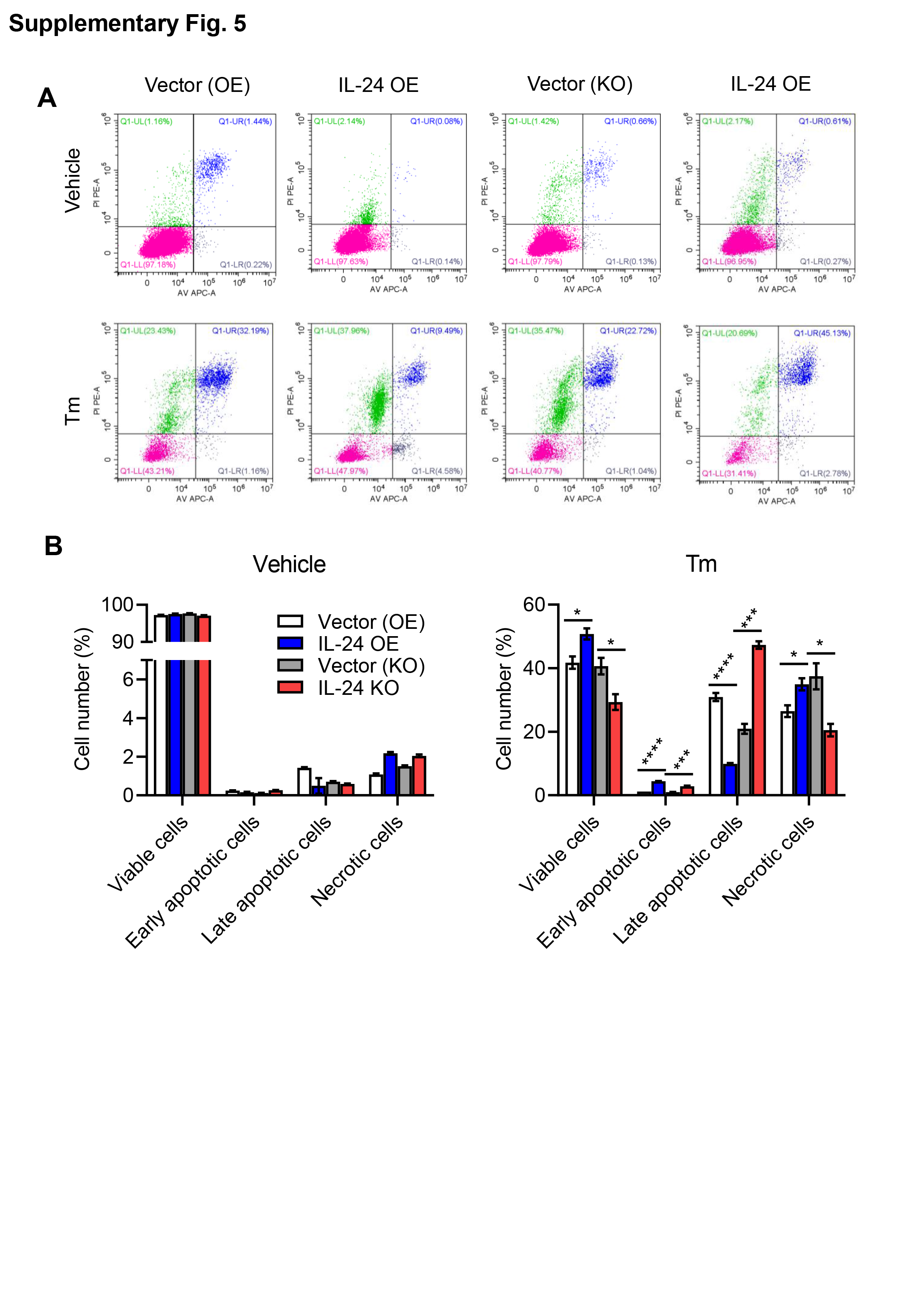

Supplement: Supplementary file 7 — Supplementary Figure 5 [file 41419_2019_2209_MOESM7_ESM.png]

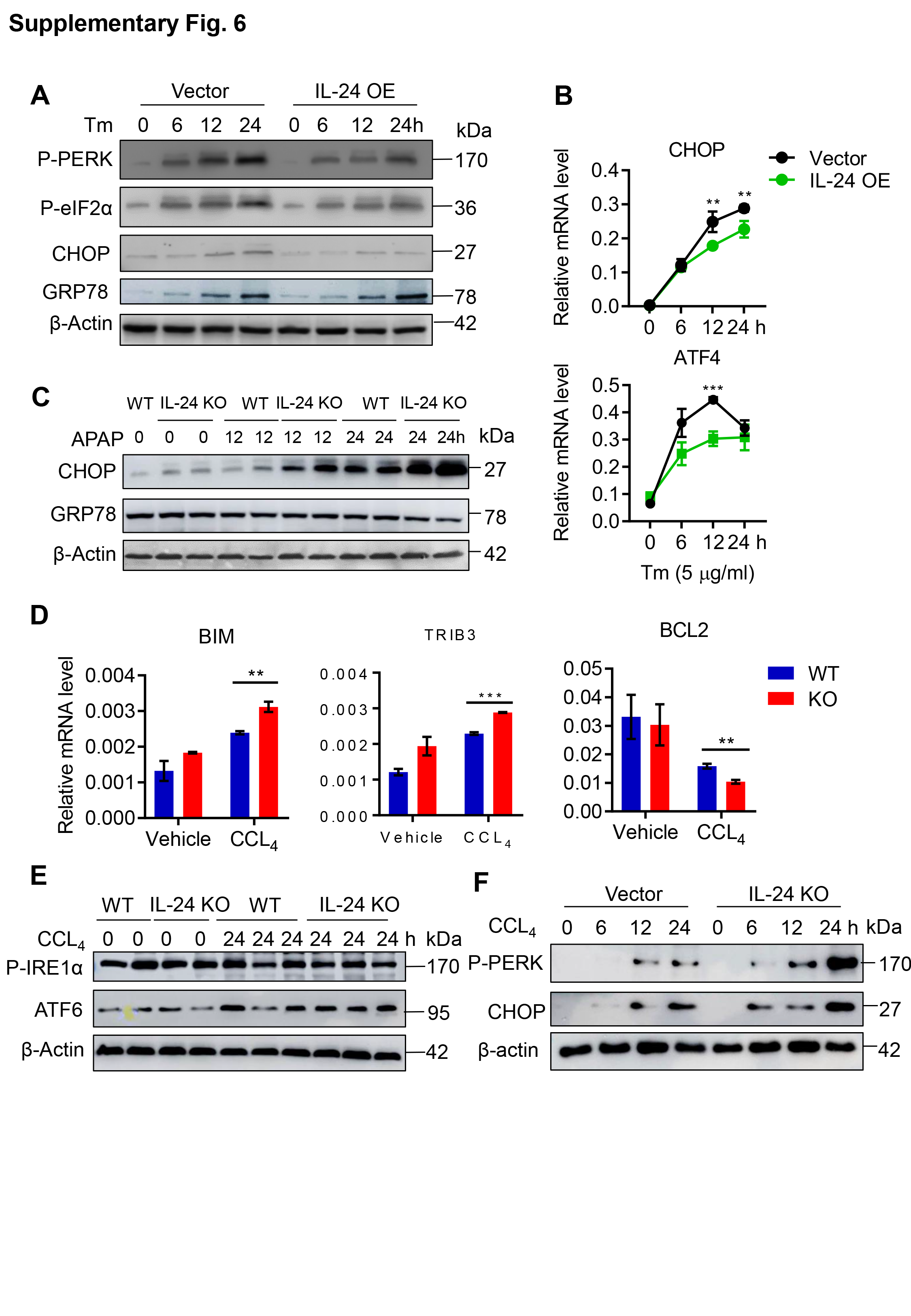

Supplement: Supplementary file 8 — Supplementary Figure 6 [file 41419_2019_2209_MOESM8_ESM.png]

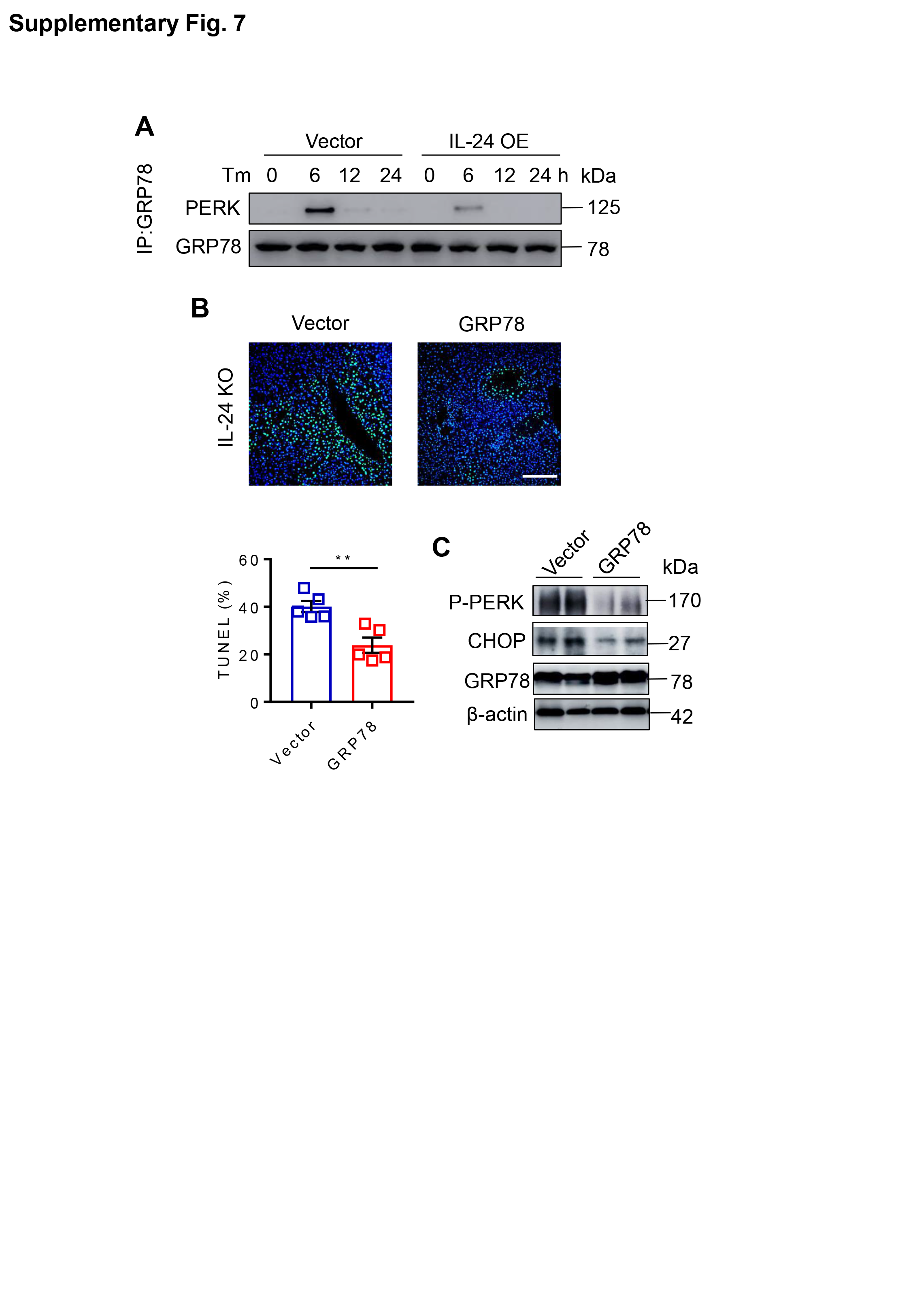

Supplement: Supplementary file 9 — Supplementary Figure 7 [file 41419_2019_2209_MOESM9_ESM.png]

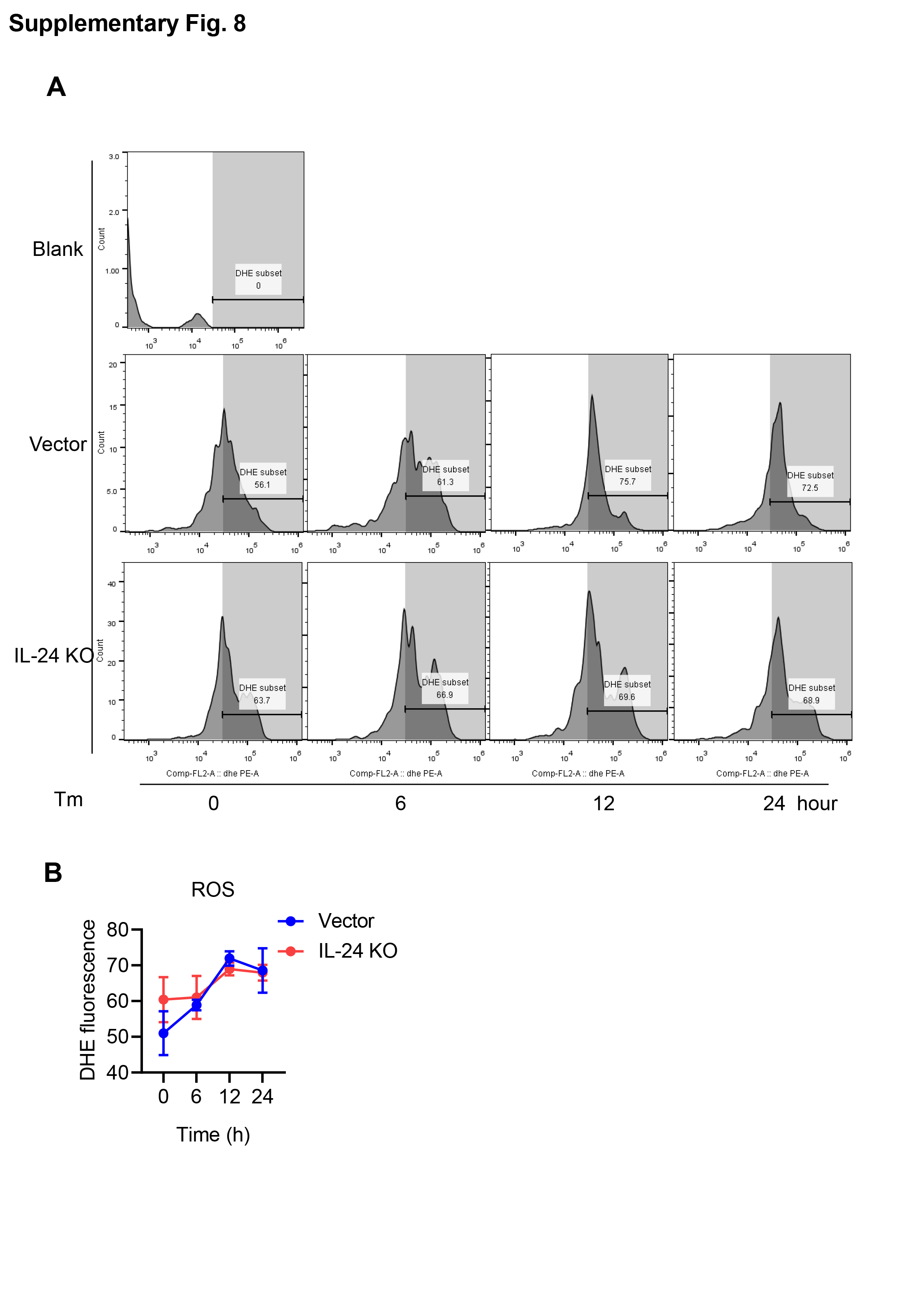

Supplement: Supplementary file 10 — Supplementary Figure 8 [file 41419_2019_2209_MOESM10_ESM.png]

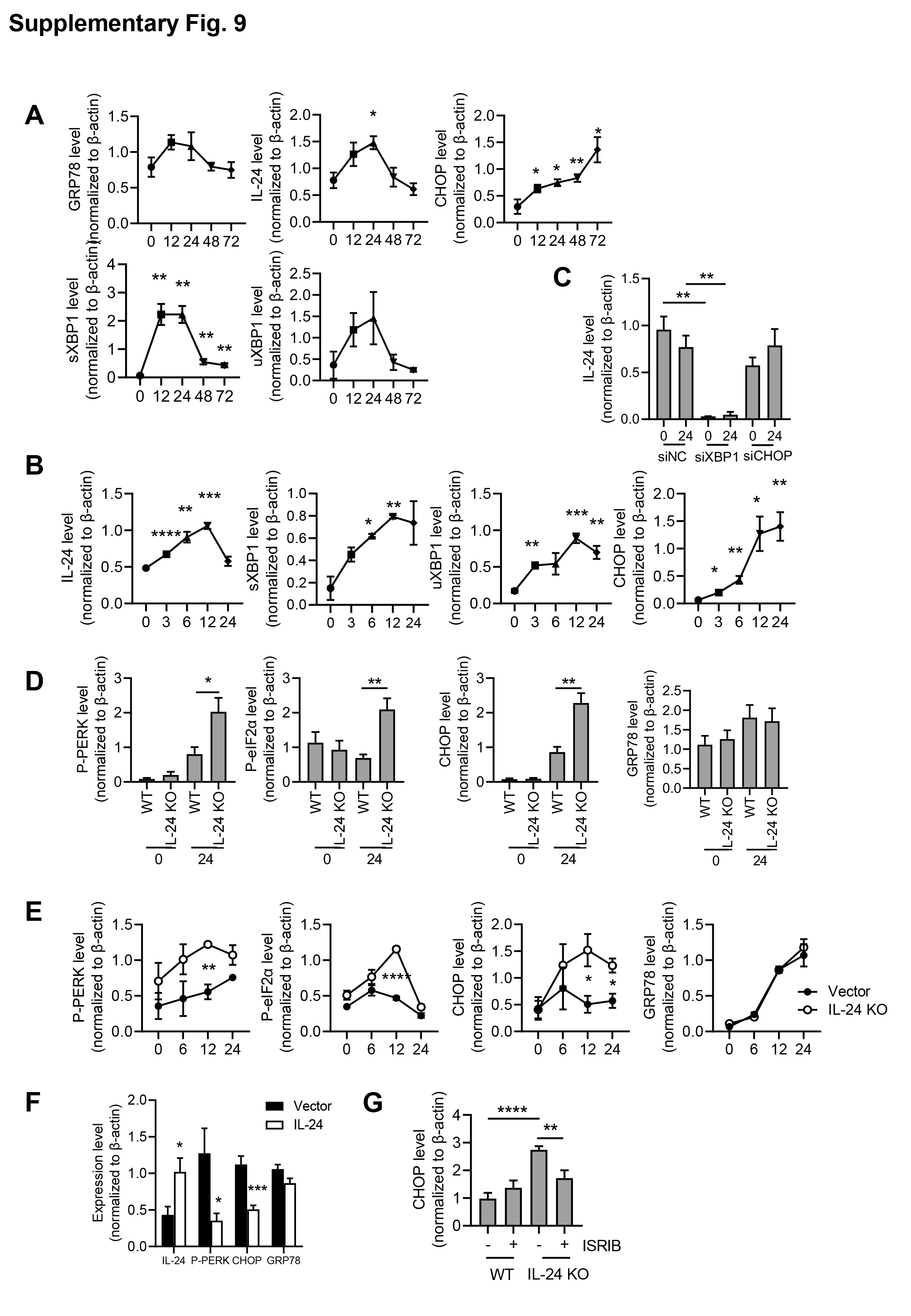

Supplement: Supplementary file 11 — Supplementary Figure 9 [file 41419_2019_2209_MOESM11_ESM.png]

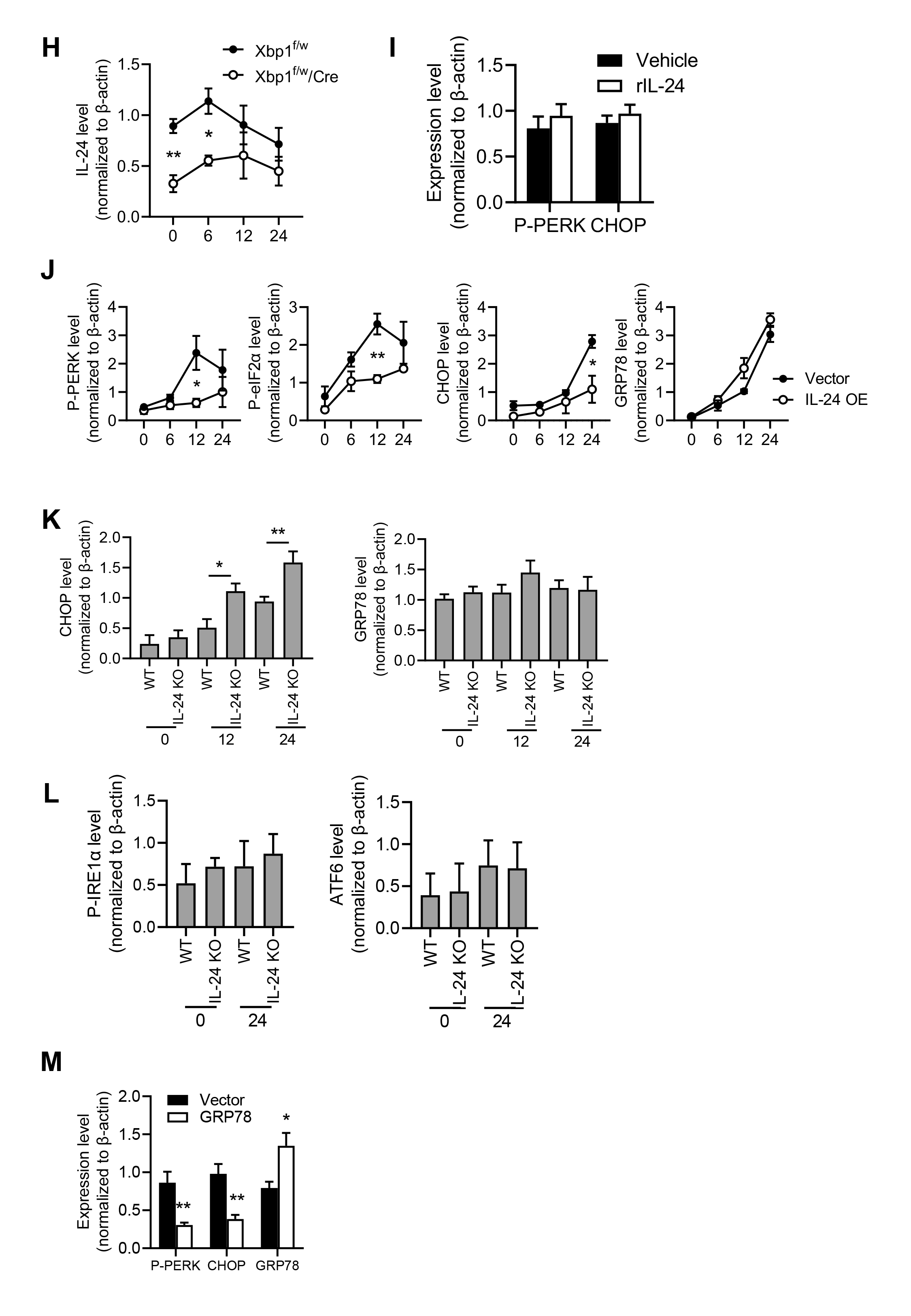

Supplement: Supplementary file 12 — Supplementary Figure 9 [file 41419_2019_2209_MOESM12_ESM.png]
