# Supplementary material for: Intracellular XBP1-IL-24 axis dismantles cytotoxic unfolded protein response in the liver
Source: Cell Death Dis. 2020 Jan 6;11(1):17. doi: 10.1038/s41419-019-2209-6 (PMC6944701; doi:10.1038/s41419-019-2209-6)
Supplement: Supplementary file 13 — Supplementary Figure Legends [file 41419_2019_2209_MOESM13_ESM.docx]

**Supplementary Fig. 1 Determination of the levels of IL-24 and UPR markers during hepatocyte stress.** (A) Representatively protein levels of IL-24 in different mouse tissues as assessed by western blot. *n* = 3 independent experiments. (B) Mouse liver function was evaluated by measuring serum ALT (left) and ­AST (right) levels 0-72 h post CCL_4_ injection. *n* = 4. (C, D) ATF4, ATF6 and sXBP1 mRNA levels in CCL_4_-treated mice (C) and Tm-stimulated AML12 cells (D), as evaluated by qRT-PCR at indicated time points. *n* = 3 biological replicates. Data are presented as means ± SEM. **P* < 0.05, ***P* < 0.01, ****P* < 0.001, *****P* < 0.0001. *P*-values were determined by two tailed *t*-test.

**Supplementary Fig. 2 Hepatocyte XBP1 is essential for maintaining IL-24 transcription.** (A) Alignment of responsive elements for CHOP (boxed) XBP1/ATF6 (red) found in the promoter region of *Il24*. The base positions of the consensus are indicated 5′→3′. Positions are relative to transcription initiation site. (B) AML12 cells were transfected with indicated siRNAs or negative control (NC) 24 h prior to Tm treatment. IL-24 mRNA levels at indicated time points were assessed by qRT-PCR. (C) *Il24* promoter activity in AML12 cells expressing siRNAs with or without Tm treatment (24 h), as quantified using luciferase assay. *Renilla* luciferase activity was normalized to firefly activity and presented as relative luciferase activity. *n* = 3 biological replicates. (D) Cell viability of Ctrl (Xbp1^f/w^) and XBP1 KO (Xbp1^f/w^Alb^Cre^) mouse hepatocytes at indicated time points post Tm treatment was assessed by CCK8 assay. *n* = 3 independent experiments. (E, F) Representatively protein (E) and mRNA (F) levels of IL-24 in Ctrl (Xbp1^f/w^) and XBP1 KO (Xbp1^f/w^Alb^Cre^) mouse hepatocytes at indicated time points post Tm treatment. *n* = 3 independent experiments (E) or biological replicates (F). Data are presented as means ± SEM. **P* < 0.05, ***P* < 0.01, ****P* < 0.001, *****P* < 0.0001. *P*-values were determined by two tailed *t*-test.

**Supplementary Fig. 3 IL-24 deficiency exacerbates ER stress-related liver injury.** (A) Representatively PCNA staining of the liver tissues from CCL_4_-treated WT and KO mice. Hepatocyte proliferation after CCL_4_-treatment was assessed by counting PCNA positive cells. Scale bar, 100 µm. (B) IL6, TNFA and IL1A mRNA levels in the liver tissues from vehicle or CCL_4_-treated WT and IL-24 KO mice. *n* = 3 mice. (C-E) Sex- and age-matched WT and IL-24-null mice were orally treated with vehicle or APAP (500 mg/kg). (C) Mouse liver function was assessed by measuring serum ALT (left) and ­AST (right) levels. *n* = 5-8. (D) Mouse survival rate after APAP-treatment was determined via Log-rank (Mantel-Cox) analysis. *n* = 9-10. (E) Representatively H&E and TUNEL staining of the liver tissues from APAP-treated WT and IL-24-null mice. *n* = 5-8. Scale bar, 100 µm. Data are presented as means ± SEM. **P* < 0.05, ***P* < 0.01, ****P* < 0.001. *P*-values were determined by two tailed *t*-test.

**Supplementary Fig. 4 Extracellular IL-24 does not affect liver function.** Recombinant IL-24 (rIL-24) (5 μg per mouse) was intraperitoneally treated one hour prior to CCL_4_ administration. (A) Mouse liver function was assessed by measuring serum ALT (left) and AST (right) levels. *n* = 6 mice. (B) Representatively H&E and TUNEL staining of the liver tissues from Vehicle or rIL-24-pre-treated mice. Scale bar, 100 μm. (C) Representatively P-PERK and CHOP protein levels in Vehicle or recombinant IL-24-pre-treated mice as evaluated by western blot at indicated time points. *n* = 3 independent experiments. (D) IL6, TNFA and IL1A mRNA levels in the liver tissues from CCL_4_-treated IL-24 KO mice with or without IL-24 re-expression. *n* = 4 mice. Data are presented as means ± SEM. ***P* < 0.01, ****P* < 0.001. *P*-values were determined by two tailed *t*-test.

**Supplementary Fig. 5 Intrinsic IL-24 reduces Tm-induced hepatocyte apoptosis.** (A, B) Ratios of early and late phases of apoptosis in AML12 cells expressing different levels of IL-24 with or without Tm treatment, as evaluated by Annexin V-PI staining. Data are presented as means ± SEM. **P* < 0.05, ****P* < 0.001, *****P* < 0.0001. *P*-values were determined by two tailed *t*-test.

**Supplementary Fig. 6** **Hepatocyte IL-24 attenuates PERK-eIF2α-CHOP branch reaction.** (A) P-PERK, P-eIF2α, CHOP and GRP78 protein levels in Tm-stimulated control and IL-24 OE AML12 cells, as assessed by western blot. *n* = 3 independent experiments. (B) CHOP (upper) and ATF4 (lower) mRNA levels in Tm-stimulated control and IL-24 OE AML12 cells, as evaluated by by qRT-PCR at indicated time points. *n* = 3 biological replicates. (C) Representatively CHOP and GRP78 protein levels in APAP-treated WT and IL-24 KO mice, as evaluated by western blot at indicated time points. *n* = 3 independent experiments. (D) Bim, TRIB3 and Bcl2 mRNA levels in the liver tissues from vehicle or CCL_4_-treated WT and IL-24 KO mice. *n* = 3 independent experiments. (E) Representatively P-IRE1α and ATF6 protein levels in CCL_4_-treated WT and IL-24 KO mice, as evaluated by western blot at indicated time points. *n* = 3 independent experiments. (F) P-PERK and CHOP protein levels in CCL_4_-treated AML12 cells, as evaluated by western blot at indicated time points. *n* = 3 independent experiments. Data are presented as means ± SEM. ***P* < 0.01, ****P* < 0.001. *P*-values were determined by two tailed *t*-test.

**Supplementary Fig. 7 GRP78 compensates the anti-stress function of hepatocyte IL-24.** (A) Immunoblotting of PERK in the precipitates obtained by immunoprecipitation of endogenous GRP78 in indicated AML12 cells. *n* = 3 independent experiments. (B) IL-24 KO mice were intravenously injected with AAV particles expressing an empty vector or mouse GRP78 8 weeks prior to CCL_4_ administration. Liver injury was assessed by TUNEL positive cell ratios. *n* = 5 mice. Scale bar, 100 µm. (C) Representatively immunoblotting of P-PERK and CHOP in the liver tissues from CCL_4_-exposed IL-24 KO mice with or without GRP78 overexpression. *n* = 3 independent experiments. Data are presented as means ± SEM. ***P* < 0.01. *P*-values were determined by two tailed *t*-test.

**Supplementary Fig. 8 The effect of IL-24 on Tm-induced ROS production in cultured AML12 cells.**

(A, B) Quantification of intracellular ROS in AML12 cells expressing different levels of IL-24 at indicated time points, as evaluated by DHE staining. n = 3 independent experiments. Data are presented as means ± SEM.

**Supplementary Fig. 9** Analyses of the band densities of (A) Fig. 1A, (B) Fig. 1C, (C) Fig. 1E, (D) Fig. 3A, (E) Fig. 3B, (F) Fig. 4E, (G) Fig. 5D, (H) Supplementary Fig. 2E, (I) Supplementary Fig. 4C, (J), Supplementary Fig. 6A (K) Supplementary Fig. 6C, (L) Supplementary Fig. 6E, and (M) Supplementary Fig. 7C.
