# Supplementary material for: Intracellular XBP1-IL-24 axis dismantles cytotoxic unfolded protein response in the liver
Source: Cell Death Dis. 2020 Jan 6;11(1):17. doi: 10.1038/s41419-019-2209-6 (PMC6944701; doi:10.1038/s41419-019-2209-6)
Supplement: Supplementary file 14 — Detailed Attribution of Authorship [file 41419_2019_2209_MOESM14_ESM.pdf]

DECLARATION OF CONTRIBUTIONS TO ARTICLE

ADMC

Manuscript Number: CDDIS-19-2272

Journal Name: *Cell Death & Disease* (the 'Journal')

Proposed Title of the Contribution:

Intracellular XBP1-IL-24 axis dismantles cytotoxic unfolded protein response in the liver (the 'Contribution)

Author(s):

Jianye Wang, Bian Hu, Zhicong Zhao, Haiyan Zhang, He Zhang, Zhenjun Zhao, Xiong Ma, Bin Shen, Beicheng Sun, Xingxu Huang, Jiajie Hou & Qiang Xia (the 'Authors')

For all *CDD* articles, each person named as an author in the published version must be able to show he or she has contributed substantially to the article.

Authorship credit should be based on 1) substantial contributions to conception and design, acquisition of data, or analysis and interpretation of data; 2) drafting the article or revising it critically for important intellectual content; and 3) final approval of the version to be published. Authors should meet conditions 1, 2 and 3.

Any person who cannot be shown to have made a substantial contribution to the article cannot be listed as an author in the final version. The name of any person who is deemed to have made a minor contribution can, however, appear in the Acknowledgments section of the article.

Please complete the table below to indicate the contributions of all named authors to the manuscript.

Author Full Name:	Specification of Contribution to the Manuscript:
Jianye Wang	contributed to conception and design, acquisition of data, analyzed and interpreted the data; drafting the article and revising it critically for important intellectual content
Bian Hu	contributed to conception and design, conducted the experiments, interpreted the data, drafted the manuscript
Zhicong Zhao	conducted the experiments, analyzed and interpreted the data, provided materials and discussion
Haiyan Zhang	provided discussion, contributions to conception and design
He Zhang	conducted the experiments, analyzed and interpreted the data, provided materials and discussion
Zhenjun Zhao	conducted the experiments, analyzed and interpreted the data, provided materials and discussion
Xiong Ma	provided discussion, contributions to conception and design
Bin Shen	provided discussion, contributions to conception and design
Beicheng Sun	provided discussion, contributions to conception and design
Xingxu Huang	conceived and designed the project, interpreted results and supervised the study
Jiajie Hou	conceived and designed the project, analyzed and interpreted results, obtained funding, wrote the manuscript, supervised the study and final approval of the version to be published
Qiang Xia	conceived and designed the project, interpreted results, obtained funding, supervised the study and final approval of the version to be published

Please complete the table below to indicate the contributions of all named authors to the figures.

Figure 1:

J.H., X.H. and Q.X. conceived and designed the experiments; J.W. designed and performed experiments; J.W. and B.H. analysed and interpreted results

Figure 2:

J.H., X.H. and Q.X. conceived and designed the experiments; J.W. and B.H. designed and performed experiments; He Zhang contributed to design and conduction of experiments and to analysis and interpretation of data; J.W. and B.H. analysed and interpreted results

Figure 3:

J.H., X.H. and Q.X. conceived and designed the experiments; J.W. and B.H. designed and performed experiments; He Zhang and Zhicong Zhao contributed to design and conduction of experiments and to analysis and interpretation of data; J.W. and B.H. analysed and interpreted results

Figure 4:

J.H., X.H. and Q.X. conceived and designed the experiments; J.W. and B.H. designed and performed experiments; Zhenjun Zhao contributed to design and conduction of experiments and to analysis and interpretation of data; J.W. and B.H. analysed and interpreted results

Figure 5:

J.H., X.H. and Q.X. conceived and designed the experiments; J.W. and B.H. designed and performed experiments; Zhicong Zhao contributed to design and conduction of experiments and to analysis and interpretation of data; J.W. and B.H. analysed and interpreted results

Figure 6:

J.H., X.H. and Q.X. conceived and designed the experiments; J.W. designed and performed experiments; He Zhang and Zhicong Zhao contributed to conduction of experiments and to analysis and interpretation of data; J.W. and B.H. analysed and interpreted results

Signed for and on behalf of the Author(s):	Print Name:	Date:
Jianye Wang [illegible]	Jianye Wang, Qiang Xia	20190929
